# Voltage-gated calcium channels act upstream of adenylyl cyclase Ac78C to promote timely initiation of dendrite regeneration

**DOI:** 10.1371/journal.pgen.1011388

**Published:** 2024-08-26

**Authors:** J. Ian Hertzler, Jiajing Teng, Annabelle R. Bernard, Michelle C. Stone, Hannah L. Kline, Gibarni Mahata, Nitish Kumar, Melissa M. Rolls

**Affiliations:** Biochemistry and Molecular Biology and the Huck Institutes of the Life Sciences The Pennsylvania State University, University Park, Pennsylvania, United States of America; University of California San Diego, UNITED STATES OF AMERICA

## Abstract

Most neurons are not replaced after injury and thus possess robust intrinsic mechanisms for repair after damage. Axon injury triggers a calcium wave, and calcium and cAMP can augment axon regeneration. In comparison to axon regeneration, dendrite regeneration is poorly understood. To test whether calcium and cAMP might also be involved in dendrite injury signaling, we tracked the responses of Drosophila dendritic arborization neurons to laser severing of axons and dendrites. We found that calcium and subsequently cAMP accumulate in the cell body after both dendrite and axon injury. Two voltage-gated calcium channels (VGCCs), L-Type and T-Type, are required for the calcium influx in response to dendrite injury and play a role in rapid initiation of dendrite regeneration. The AC8 family adenylyl cyclase, Ac78C, is required for cAMP production after dendrite injury and timely initiation of regeneration. Injury-induced cAMP production is sensitive to VGCC reduction, placing calcium upstream of cAMP generation. We propose that two VGCCs initiate global calcium influx in response to dendrite injury followed by production of cAMP by Ac78C. This signaling pathway promotes timely initiation of dendrite regrowth several hours after dendrite damage.

## Author summary

To last a lifetime, neurons must have robust stress and injury response pathways. While axon regeneration has long been appreciated as an important neuronal repair pathway, dendrite regeneration has only recently emerged as a potential contributor to neuronal health. The first step in mounting an injury response is detecting the injury. We ask whether axonal injury signals calcium and cAMP also play a role in sensing dendrite injury. We show that, although axon and dendrite injury trigger different growth pathways, they both elicit similar global calcium and cAMP elevation in the minute following injury. We identify the proteins responsible for this early calcium and cAMP rise in response to dendrite injury and show that they are important for rapid initiation of dendrite outgrowth. This characterization of early events in the dendrite injury response is an important foundation for understanding how dendrite regeneration works and how different responses are triggered by axon and dendrite injury.

## Introduction

The ability of individual neurons to mount repair programs after injury is critical for long term nervous system function. Axon regeneration in the vertebrate peripheral nervous system allows recovery of function after nerve damage [1,2,3,4] and has been intensively studied for over a hundred years. More recently, axon regeneration has been detected in invertebrate model systems, and genetic studies in *Drosophila* and *C*. *elegans* have provided key insights into conserved signaling pathways that activate axon regeneration [5,6,7,8,9]. In contrast, dendrite regeneration was first detected in *Drosophila* [10,11], and more recently was shown to occur in the vertebrate central nervous system [12]. Very little is known about signals generated by dendrite injury that initiate regeneration [13].

The most general cellular injury signal is rapid calcium influx through damaged plasma membrane, and this is required for rapid closure of the membrane breach [14,15,16]. Calcium has additional roles in the axon injury response. In cultured *Aplysia* neurons calcium at the injury site helps establish a new growth cone within minutes [17] and in mammalian peripheral neurons local calcium helps remodel microtubules near the injury site to facilitate growth [18]. Calcium signals also propagate away from the axon injury site to activate more global signaling. A wave of high calcium moving away from the damaged axon towards the cell body has been visualized in *C*. *elegans* [19] and mammalian [18,20,21] neurons. This wave was shown to reach the cell body in rodent neurons [18,21]. Voltage-gated calcium channels (VGCCs) are required for the broad injury-induced calcium increase [19,20,22], and blocking VGCCs reduces regeneration in *C*. *elegans* [19] and cultured sympathetic neurons [23]. Calcium release from the endoplasmic reticulum (ER) may contribute to the early calcium wave [19,20], but has also been proposed to function locally in the growth cone at a later step [24].

Another second messenger linked to axon regeneration is cyclic AMP (cAMP). In 1972, cAMP addition to dorsal root ganglion explants was shown to enhance axon outgrowth [25] and cAMP levels were found to increase in sciatic nerves after crush [26]. Pre-injury levels of cAMP were later correlated with ability of axons to regenerate onto inhibitory substrates [27] and exogenous cAMP was found to promote axon regeneration in the CNS, where regeneration is normally poor [28,29]. Moreover, genetically increasing cAMP levels in *C*. *elegans* and *Drosophila* increases axon growth in the first day after injury [19,30].

It has been suggested that the early calcium rise that occurs seconds to minutes after injury activates adenylyl cyclase to generate cAMP [2,19]. However, the only studies to show that cAMP levels increase in response to axon injury have been performed on the hours timescale and agree that cAMP levels proximal to the injury site are elevated 24 hours after damage [26,29]. One of the studies analyzed earlier time points and found no change [26]. The timing of the reported axonal cAMP increase is consistent with increased adenylyl cyclase activity in ligated nerves 8–24 hours after injury [31], but not consistent with being an output of the initial calcium increase. The relationship between early increases in calcium and the effect of cAMP on axon regeneration therefore remains unclear. A specific adenylyl cyclase responsible for increasing cAMP in response to axon injury has not been identified.

It has recently been shown that dendrite damage can trigger regenerative outgrowth in invertebrate [10,11,32] and vertebrate [12] model systems. Moreover, regeneration of sensory dendrites in Drosophila [33] and *C*. *elegans* [34] allows function to recover. Signals that initiate dendrite regeneration have not been identified. As calcium is a very general injury signal and cAMP has been linked to outgrowth induced by axon injury, we wished to test whether these second messengers might be involved in the response to dendrite injury.

Drosophila sensory neurons are the best-established system in which to study dendrite regeneration [13]. The highly branched dendrites of nociceptive neurons (Class IV dendritic arborization neurons) innervate the Drosophila larval epidermis [35,36]. After removal of the entire dendrite arbor, dendrites regrow to cover their territory within 96 hours [11]. Growth initiates very rapidly and by 24 hours after injury an arbor about 200 microns in diameter has extended [37]. Also by 24 hours after injury the neurons regain competency to trigger escape behavior with similar efficiency to uninjured neurons [33]. We therefore chose to investigate calcium and cAMP injury signaling in these cells using laser microsurgery. We first confirmed that, as in *C*. *elegans* and mammalian neurons, axon injury of the nociceptive ddaC neuron results in a calcium transient on the seconds time scale. Dendrite injury elicits a calcium increase of similar magnitude. VGCCs Ca-α1D (L-type) and Ca-α1T (T-type) were both required for the response after dendrite injury. To determine whether injury causes a rapid increase in cAMP, we monitored it using a genetically encoded sensor. Both axon and dendrite injury increased cAMP, with levels in the cell body peaking about a minute after transection. Moreover, VGCC knockdown reduced the increase in cAMP after dendrite injury demonstrating that calcium influx in response to dendrite injury acts upstream of cAMP production. To test functional importance of calcium, we examined dendrite regeneration in VGCC knockdown backgrounds. Initiation of growth was delayed, consistent with a role for calcium in early injury signaling. To determine whether cAMP is a key effector of calcium after dendrite injury we performed a candidate screen to identify the adenylyl cyclase that acts in response to dendrite severing. We found that the AC8 family member, Ac78C, is required for cAMP production after dendrite injury and, like VGCCs, is required for timely initiation of regrowth. We conclude that calcium entry through VGCCs acts upstream of Ac78C to help initiate dendrite outgrowth after injury.

## Results

### Axon and dendrite injury cause similar calcium increases in the neuronal cell body

**Fig 1:**
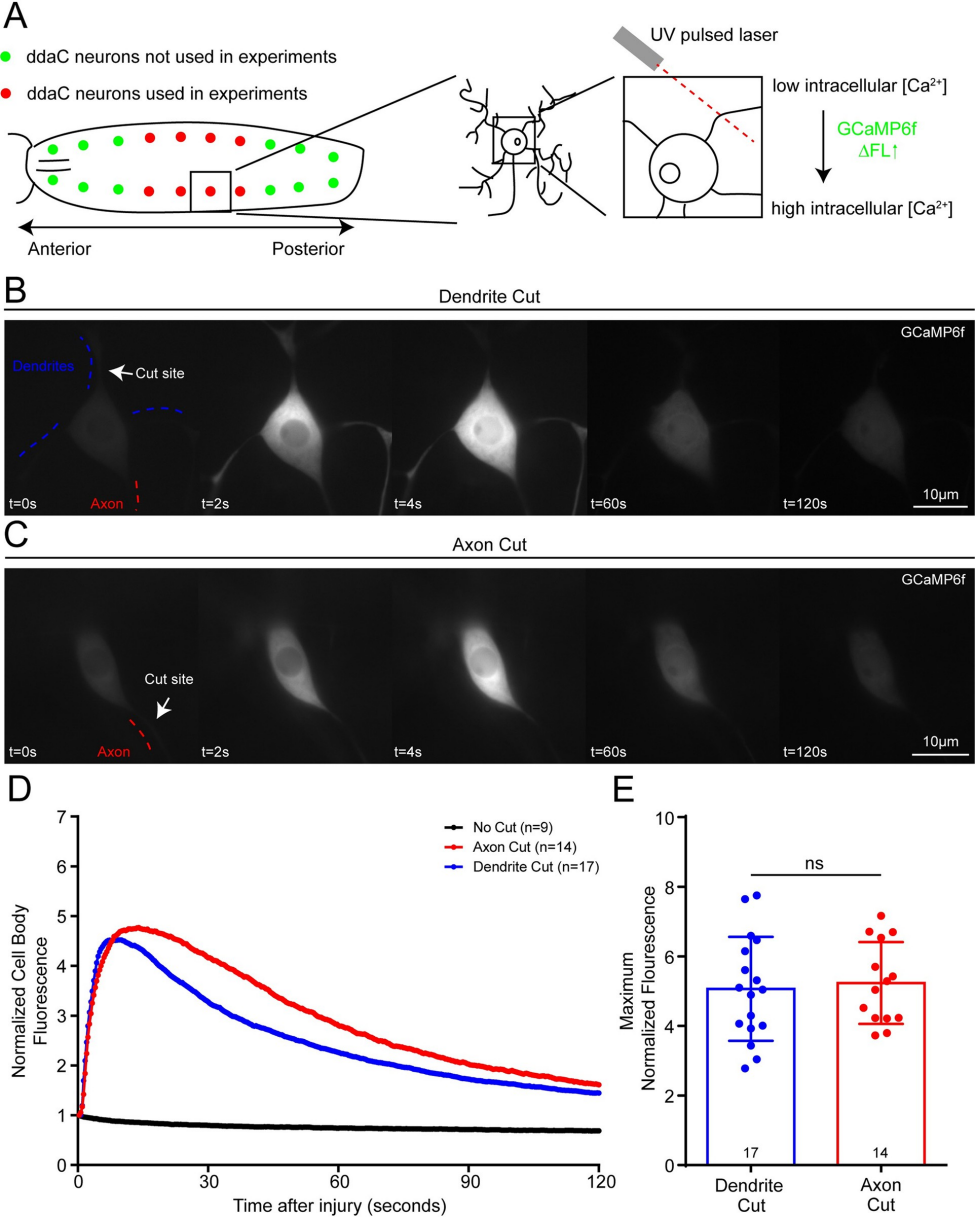
Calcium influx during axon and dendrite injury to class IV (ddaC) neurons. (A) Diagram of location of ddaC neurons in larvae and location of laser cuts of the neurons. We severed either axons or dendrites of ddaC neurons with a UV laser and monitored calcium levels in the soma simultaneously with the GCaMP6f calcium sensor. Example time series of GCaMP6F fluorescence during dendrite (B) and axon (C) injury. Cut site is labeled with a white arrow. Axonal compartment is labeled with a red dashed line, and the dendritic compartments are labeled with blue dashed lines. GCaMP6f fluorescence increased 4–5 fold within 10–15 seconds after injury. Average normalized fluorescence intensities of the cell body are shown (D). The peak fluorescence of each injured neuron is graphed (E), and this is not different between axon and dendrite injuries with a Mann-Whitney test. Error bars for (D) are omitted for clarity, but relevant error information is contained in (E), where bars represent standard deviation.

Axons and dendrites contain different sets of channels to allow for integration of electrical signals in dendrites and transfer of signals over long distances in axons. As global calcium elevation in response to axon injury involves VGCCs, and some VGCCs function specifically in axons, it is not clear whether dendrite injury also results in global calcium increases. To assess whether calcium could alert the cell body to dendrite damage, we imaged Drosophila ddaC (class IV) nociceptive neurons expressing GCaMP6f [38] while axons or dendrites were severed with a pulsed UV laser (Fig 1A). The standard injury paradigm for axons involves severing axons tens of microns to millimeters from the soma, while most studies on dendrite injury remove dendrites near the cell body. Because we were particularly interested in signals that initiate dendrite regeneration, we chose to injure dendrites near the soma as this very reliably elicits regeneration. For comparison purposes, we also injured axons near the soma. GCaMP6f fluorescence in the cell body was strongly increased on the seconds time scale in response to axon injury (Fig 1C and 1D and S1 Movie). A response of similar magnitude was seen after dendrite injury but decayed slightly faster than after axon injury (Fig 1B, 1D, and 1E and S2 Movie). After both types of injury, the somatic calcium increase was accompanied by increased GCaMP6f signal in dendrites (Figs 1B and S1 and S1 and S2 Movies). When we examined the first few frames in more detail, there was often a slight global increase in GCaMP fluorescence in the first frame (S1 Fig). This was then followed by a wave of higher fluorescence sweeping across the cell body from the base of the injured neurite. The wave was more visible in some neurons; several examples where it could be clearly observed are shown in S1 Fig. After the initial wave, fluorescence remained evenly high in the cell body and then gradually diminished (S1 and S2 Movies). We did not detect any distinct calcium entry at the wound site that was separable from the wave that spread away from it (Figs 1 and S1 and S1 and S2 Movies). Importantly, the peak did not represent saturation of GCaMP6f. In this study, as in our previous characterization of axon and dendrite injury responses [11,39,40] we attenuated the pulsed UV laser to the point where it could just sever the neurite. We have previously observed that using higher laser power can cause what we term an “explosion cut”. This is distinguished by a bright flash likely due to generation of a laser cavitation bubble, a type of injury that has been associated with cellular calcium entry even at large distances from the injury site [41]. Indeed, explosion type cuts adjacent to the dendrite (sham) caused calcium elevation in the neuron, while the type of “soft” cuts we use routinely did not (S2 Fig). When we used GCaMP6f to monitor calcium after explosion injury, its fluorescence increased higher than after our standard axon or dendrite injury (S2 Fig) indicating that the sensor is not saturated under normal injury conditions.

In these experiments we severed the axon or a single dendrite. Although removing a single dendrite can elicit regeneration in Drosophila nociceptive neurons [10], most studies use complete arbor removal [11,42]. To test whether performing multiple cuts on the same cell would lead to different calcium dynamics in the cell body, we compared GCaMP6f fluorescence after cutting a single dendrite or all dendrites (S3A Fig). Peak fluorescence and decay time were similar in both scenarios (S3B Fig). The apparently earlier peak after cutting all dendrites was due to the time needed to complete the additional injuries before starting to image. Previous studies on calcium entry after axon injury damaged the axon more distally [18,19,20,21], so we wished to compare calcium effects of proximal injury to more distal ones. While both proximal and distal axon injury can lead to regeneration, injury closer than about 50 microns from the cell body favors growth of a new axon from a dendrite, while more distal injury favors regrowth from the cut stump in Drosophila and rodent neurons [40,43]. Increasing the injury distance from within 10 microns of the cell body to 40 microns reduced the amplitude of GCaMP6f signal in the cell body, and this reduction was stronger in dendrites than axons, but peaks were still present (S3C–S3E Fig). At 80 microns from the cell body, dendrite injury led to a much smaller somatic peak (S3D Fig). We conclude that both axon and dendrite injury lead to calcium elevation in the neuronal cell body, although this is reduced when dendrites are injured far from the cell body.

### Voltage-gated calcium channels mediate calcium influx during injury

There are two main ways calcium levels could enter the cytosol at sites distant from the injury: efflux from endoplasmic reticulum (ER) stores, or influx via VGCCs. Release of calcium from the ER is through the inositol trisphosphate receptor (IP3R) or the ryanodine receptor (RyR), and both have been linked to the axon injury response [19,24]. Depletion of ER calcium stores can lead to opening of the store-operated calcium entry (SOCE) channel composed of ER calcium sensor Stim and plasma membrane channel Orai [44], and this could help sustain high levels of cytosolic calcium. To test whether ER-derived calcium or SOCE was important for the response to axon or dendrite injury, we used the Gal4-UAS system to express hairpin RNAs in nociceptive neurons. This method of cell type-specific RNAi is extremely useful for investigating the function of genes, including essential genes, in specific cells in whole animals [45]. We saw no significant difference between the peak GCaMP6f values of control, RyR, IP3R, Stim or Orai RNAi neurons after dendrite or axon injury (S4A–S4C Fig). We then tested whether these RNAi knockdowns affected dendrite regeneration. We assayed new arbor growth 24 hours after removal of all dendrites from the ddaC neuron as this approach has previously proven useful for identifying genes involved in dendrite regeneration [37]. In most cases we used two different RNA hairpins, labeled A and B (S4D Fig) to knock down targets. We did not find any condition that significantly reduced growth (S4D and S4E Fig). Because we do not know how complete knockdown is using these hairpins, we also used an imaging approach to test whether calcium was released from the ER. ER-GCaMP6-210 is a low affinity version of GCaMP targeted to the ER lumen [46] that detects physiologically relevant changes in ER calcium in Drosophila neurons [47]. If the ER was the source of the cytosolic calcium detected in response to axon or dendrite injury, then fluorescence of ER-GCaMP6-210 would be expected to decrease in the seconds after injury. We did not detect any decrease that could not be accounted for by photobleaching after severing axons or dendrites (S5 Fig). Only positioning the pulsed UV laser within the cell body resulted in decreased fluorescence (S5 Fig). Using these methods, we did not find evidence that ER-derived calcium contributes to axon or dendrite injury-induced increases in somatic calcium. We therefore moved on to testing VGCCs.

**Fig 2:**
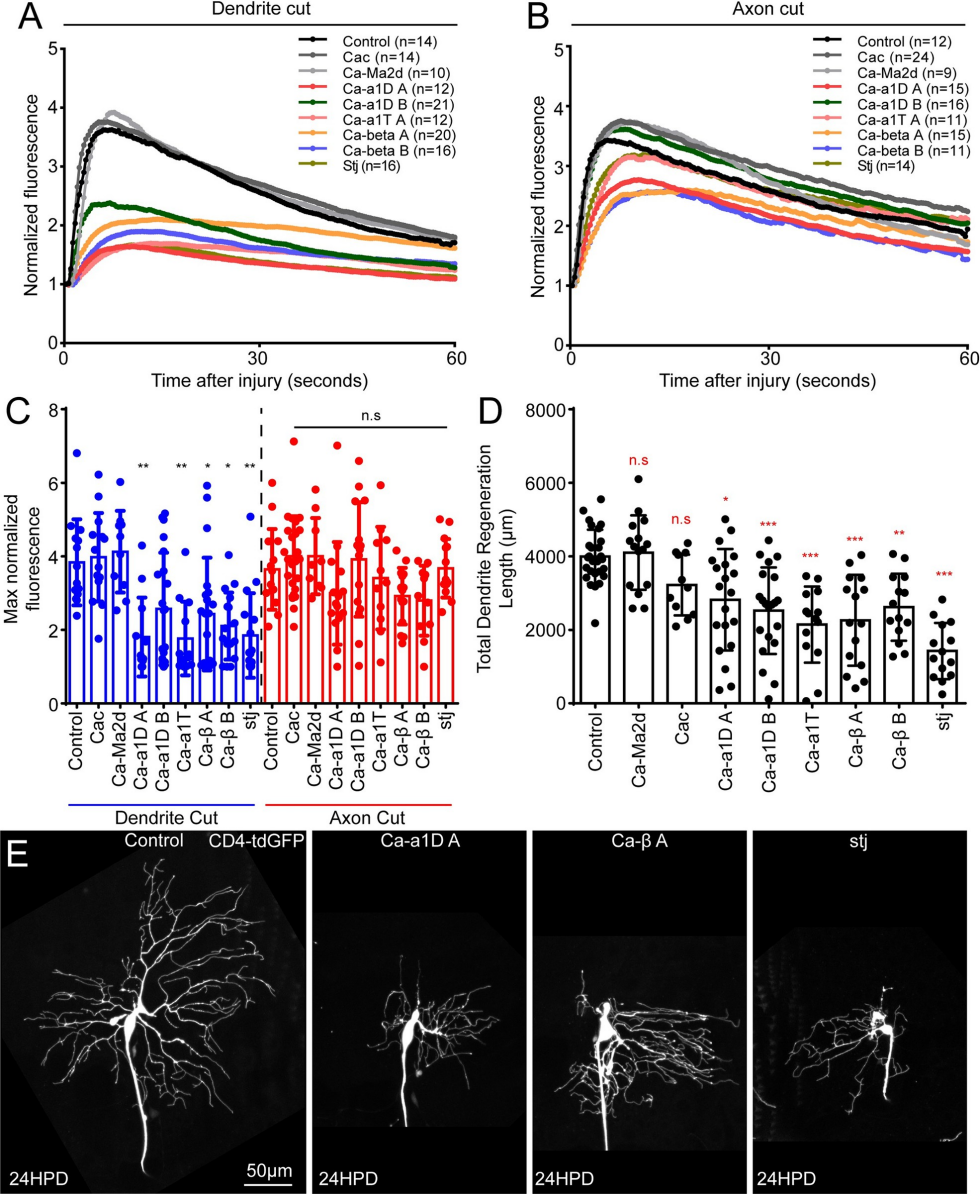
Voltage-gated calcium channels (VGCCs) are required for injury-induced calcium influx and dendrite regeneration. We performed GCaMP6f live imaging while knocking down VGCC subunits with RNAi in ddaC neurons (see Reagent table (S2 Table) for specific tester line and RNAi lines). Average GCaMP6f fluorescence is plotted over time for each genotype after dendrite injury (A) and axon injury (B). Peak fluorescence values are plotted in (C). Peak values of Ca-α1D A, Ca-α1T, Ca-β and stj RNAi neurons are significantly lower than control for the dendrite cut but not axon cut condition. (D) Total dendrite regeneration length at 24HPD is shown for each RNAi line used. (E) Example dendrite regeneration images are shown 24h after dendrite removal. All statistics are Kruskal-Wallis one way analysis of variance (ANOVA), with Dunn’s multiple comparisons test. *, p<0.05, **, p<0.01, ***, p<0.001. Error bars in (C) and (D) are standard deviations. Error bars are omitted in (A) and (B) for clarity, but relevant error information is contained in (C).

VGCCs contain a large α1 subunit that has 24 transmembrane domains and makes up the channel pore, as well as accessory β and α2δ subunits [48]. The Drosophila genome contains three alpha subunits (cac, Ca-α1D and Ca-α1T), three characterized α2δ subunits (stj, stol and Ca-Mα2δ), and one beta subunit (Ca-β) [49,50,51]. The three alpha subunits in Drosophila represent the three major classes of VGCCs in animals. Cac is the N/P/Q or Ca_v_2 representative, Ca-α1D is the L-type or Ca_v_1 representative, and Ca-α1T is the T-type or Ca_v_3 channel. In general, L-type calcium channels are activated at relatively high voltages and tend to inactivate slowly, while T-type channels are activated at more negative voltages and inactivate rapidly and the N/P/Q types are more variable and tend to be more neuron-specific [48]. Ca-α1T has been shown to be a low-voltage activated channel in a motor neuron [52] and to mediate a transient calcium current in a projection neuron [53] consistent with it behaving like T-type channels characterized in other organisms. Cac localizes to neuromuscular junctions [54,55] and is responsible for calcium-dependent neurotransmitter release [56]. The L-type channel Ca-α1D is the only one previously characterized in dendritic arborization sensory neurons; it has been shown to propagate mechanical signals through the Class IV neuron dendrite arbor [57], to mediate calcium transients in Class IV dendrite branches prior to pruning [58], and to mediate global calcium transients during axon regeneration of Class IV neurons [59]. We used cell type-specific RNAi to reduce each of the alpha subunits as well as two of the α2δ subunits and the beta subunit in neurons that expressed GCaMP6f. When we used two different RNAi transgenes to target the same gene they are labeled A and B. We found RNAi hairpins targeting both Ca-α1D and Ca-α1T, as well as accessory subunits Ca-β and stj, significantly reduced the peak of the calcium influx occurring after dendrite injury (Fig 2A and 2C and S3 Movie). To try to determine if we could distinguish between Ca-α1D and Ca-α1T roles in the calcium increase we scored each movie based on whether we could detect a wave of high calcium moving across the cell body. In control neurons about 80% of cells exhibited a detectable wave after injury, with the remaining increasing in fluorescence globally at the same time (S6 Fig). In Ca-α1D and Ca-α1T RNAi, about half of the neurons did not have a detectable increase in calcium and the other half still had a wave of signal spreading across the cell body, albeit dimmer that in control neurons (S6B Fig). After axon injury, both Ca-α1D and Ca-α1T RNAi reduced the number of neurons with a wave type increase (S6B Fig). Therefore, we could not determine whether Ca-α1D and Ca-α1T contribute in different ways to the somatic injury-induced calcium.

The other two channel subunits, Cac and Ca-Mα2δ did not affect dendrite injury-induced calcium elevation in the cell body (Fig 2A–2C) consistent with Cac function at the synapse [54] and Ca-Mα2δ in muscle [60]. No significant effects on the magnitude of calcium entry in response to axon injury were observed (Fig 2B and 2C). This may reflect robustness of the response to axon injury to partial knockdown of VGCCs, perhaps because of the ability of the axon initial segment in these cells to cluster channels [61].

To address whether calcium influx in response to dendrite injury was functionally important, we performed a laser-mediated cut at the base of every dendrite in VGCC knockdown ddaC neurons and assayed outgrowth of new dendrites 24 hours later. Each RNAi hairpin that reduced the peak calcium response to dendrite severing also reduced the total length of the new dendrite arbor (Fig 2D and 2E). These results indicate that a major pathway for calcium entry to the cell body cytoplasm after dendrite damage is through VGCCs. Moreover, VGCCs are required for robust outgrowth of a new arbor in the day after its removal.

### Injury-induced calcium influx promotes initiation of dendrite regeneration

**Fig 3:**
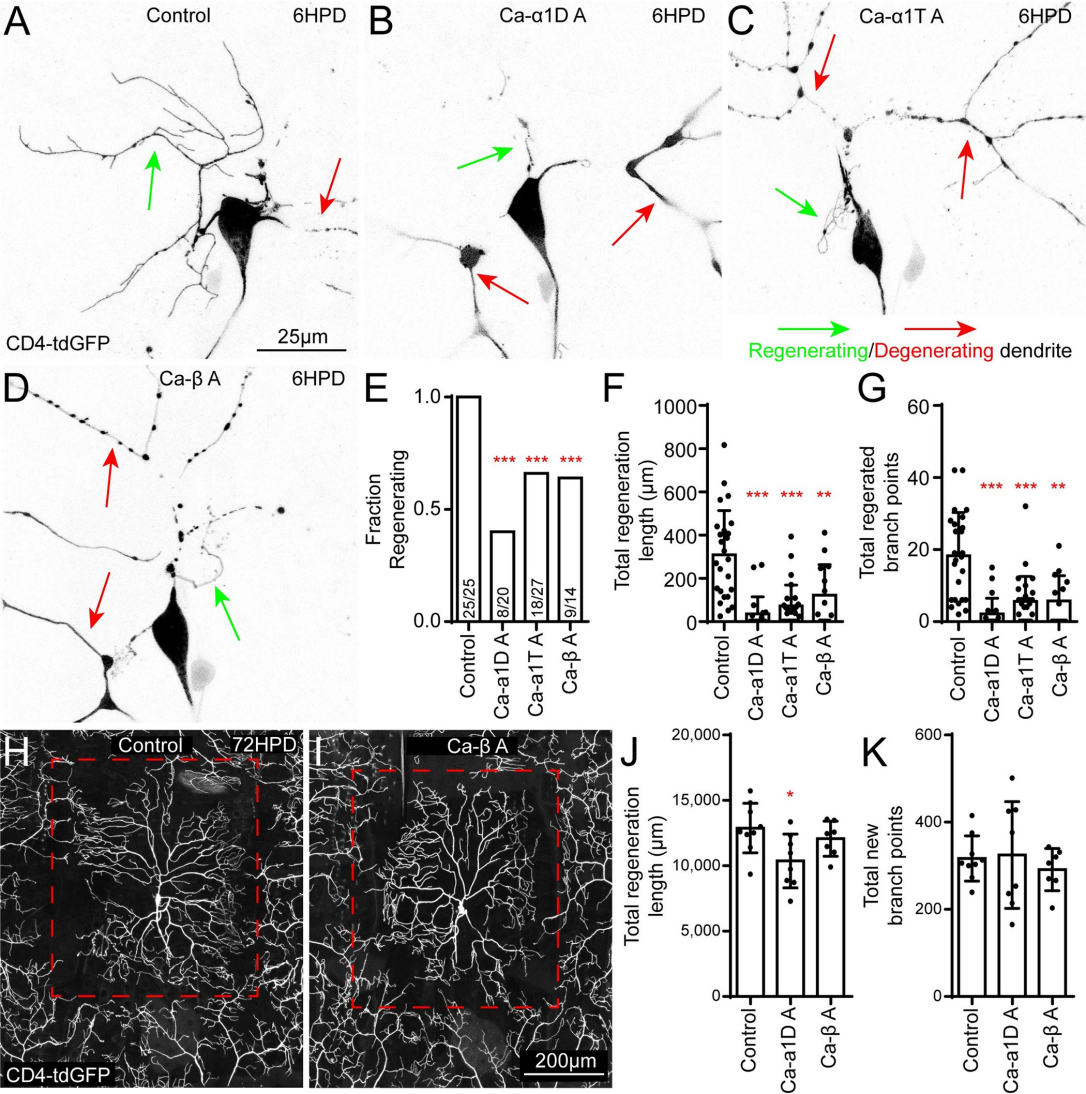
VGCCs are required for early initiation of dendrite regeneration. We assayed ddaC dendrite regeneration at 6HPD and 72HPD in control and VGCC RNAi neurons. Example images are shown for Control (A) and VGCC subunit RNAi (B-D) conditions. Green arrows highlight new dendrites and red arrows show remaining degenerating dendrites. (E) The fraction of neurons with at least 1 new dendrite branch at 6HPD is shown. All three RNAi conditions are significantly lower than control with individual Chi-square tests. Total regeneration length and total regenerated branch points at 6HPD are quantified in (F) and (G) and compared with a Kruskal-Wallis one way analysis of variance (ANOVA), with Dunn’s multiple comparisons test. (H) We assayed dendrite regeneration with these same RNAi lines at 72HPD and found little difference between conditions; example images for control and Ca-β RNAi are shown (H and I). Red dashed lines show the approximate area originally occupied by dendrite. Total regeneration length and total regenerated branch points 72HPD are quantified in (J) and (K) and both are compared with Kruskal-Wallis one way analysis of variance (ANOVA), with Dunn’s multiple comparisons test. For all statistics, *, p<0.05, **, p<0.01, ***, p<0.001. Error bars are standard deviations.

A partial reduction in dendrite regeneration 24 hours after injury with VGCC knockdown suggests that either dendrites grow more slowly, or they initiate outgrowth later than control cells. To distinguish between these two possibilities, we assayed neurons at different times after injury. By 6 hours after injury all control neurons had started regenerating fine processes (Fig 3A and 3E) that on average measured 300 μm (Fig 3F) and included almost 20 branch points (Fig 3G). In contrast, when we knocked down Ca-α1D, Ca-α1T, and Ca-β fewer neurons started to regenerate (Fig 3B–3E), and those that did had short, sparse arbors (Fig 3F and 3G). This early reduction in outgrowth suggests VGCCs are important for initiation of regeneration. One potentially confounding factor at this early time point is debris remaining from cut off dendrites. It is not known whether dendrite debris might repel or inhibit new dendrites from growing into the area. We therefore scored images from each genotype based on whether they had no remaining debris, beaded debris, or regions with continuous debris (S7A–S7C Fig). There was some variability in degeneration across genotypes (S7E Fig), so we wished to test more directly whether slowing degeneration would inhibit regeneration. Dendrite degeneration is strongly delayed by expression of the Wallerian degeneration slow (Wlds) transgene [62]. We therefore expressed Wlds in ddaC neurons and scored degeneration and measured regeneration 6 hours after dendrite removal. As expected, cut off regions of dendrites remained continuous in Wlds-expressing neurons (S7D and S7E Fig). However, regeneration still initiated, with new dendrites often growing over remaining severed dendrites (S7D Fig). No significant difference in regeneration length or branch number was found between control and Wlds-expressing neurons (S7F and S7G Fig) suggesting that delayed degeneration does not strongly affect initiation of dendrite regeneration. This finding supports the role of VGCCs in initiation of dendrite regeneration, rather than influencing regeneration by a change in rate of degeneration.

By 24 hours after injury most VGCC RNAi neurons had begun to grow new dendrites suggesting that regeneration is delayed rather than eliminated in these genetic backgrounds. To test whether VGCCs might also play a role in later stages of regeneration, we assayed regrowth at 72 hours after dendrite removal. At this time arbors of Ca-α1D and Ca-β neurons were almost indistinguishable from control arbors (Fig 3H–3K). To further test the specificity of their function in early stages of dendrite regrowth, we imaged nociceptive (Class IV) sensory dendrites in the adult abdomen. A subset of larval dendritic arborization neurons survives into adulthood to innervate the body wall. During pupariation the larval dendrite arbor is pruned and new dendrites grow into the remodeled body wall [63,64]. We imaged Class IV dendritic arborization neurons in abdomens of young adult flies expressing control or VGCC RNA hairpins. In all genetic backgrounds, the abdomen was covered in branched sensory endings (S8 Fig). We conclude that VGCCs help promote early initiation of injury-induced dendrite regeneration, but do not seem as important for later stages of dendrite growth.

### Neurons with simple arbors also use VGCCs to initiate dendrite regeneration

**Fig 4:**
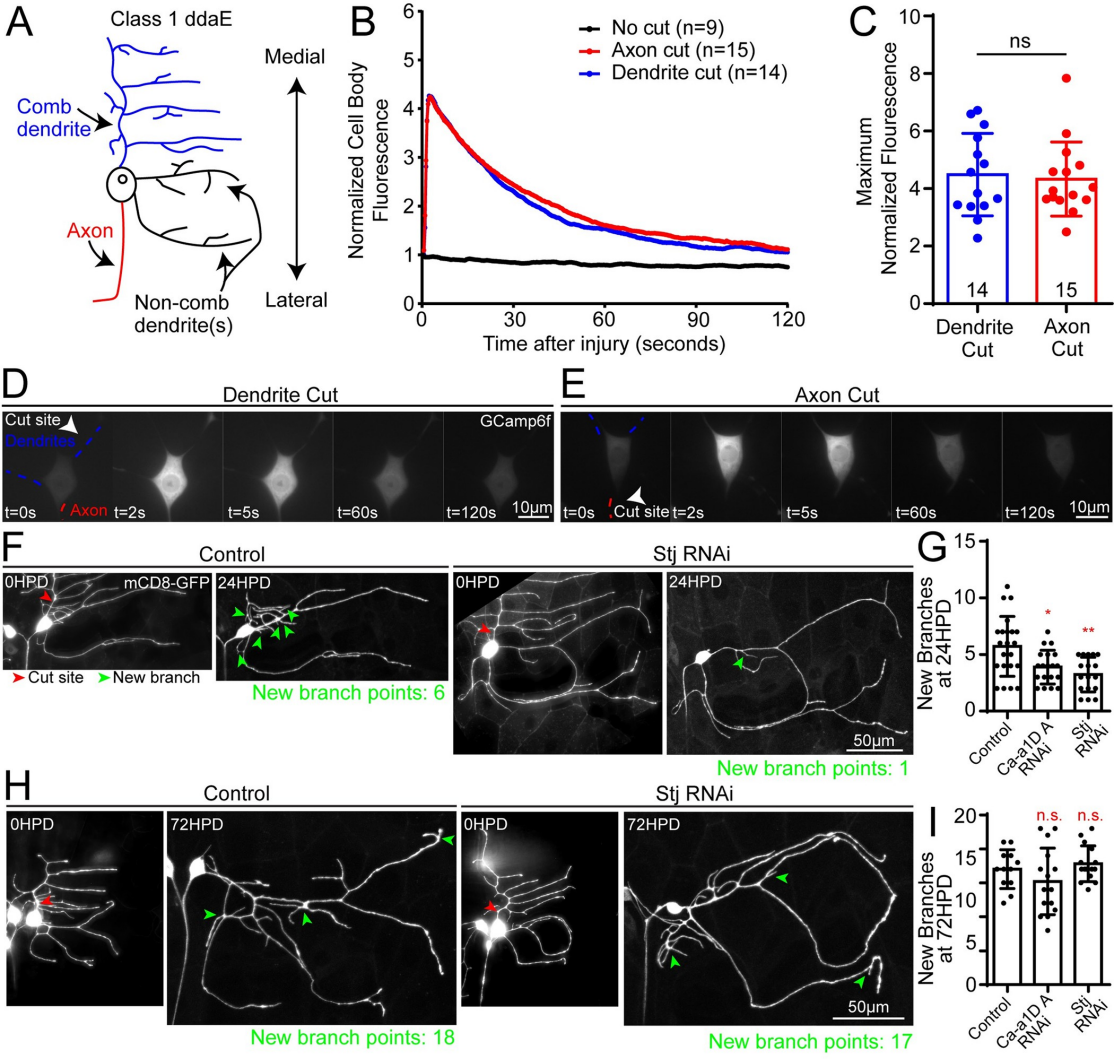
Class I (ddaE) neurons require VGCCs for dendrite injury signaling. We performed dendrite and axon cuts in ddaE neurons expressing GCaMP6f. (A) Diagram of a ddaE neuron. (B) Average GCaMP6f fluorescence intensity in the cell body over time after injury is shown for dendrite (blue) and axon (red) cuts. Peak fluorescence values are plotted in (C) and are not significantly different when compared with a Mann-Whitney test. (D and E) Blue and red dashed lines show dendrites and axons respectively, while white arrows denote location of laser severing. Example time series images are shown for 0, 2, 5, 60, and 120 seconds post cut. (F) The dorsal comb dendrite of ddaE neurons was severed (red arrow) at time 0 and regeneration was assayed by counting added branch points (green arrows) to remaining dendrites 24HPD. (G) Number of added branches at 24HPD is quantified for control, Ca-α1D and Stj RNAi genotypes. Both Ca-α1D and Stj RNAi regenerated significantly fewer branch points than control. The statistical test used was a Kruskal–Wallis one-way analysis of variance (ANOVA) with Dunn’s multiple comparisons test. *, p<0.05, **, p<0.01. All error bars are standard deviations. (H and I) A regeneration assay similar to that in F and G was performed, except that instead of assaying added branches at 24 HPD, the branches were counted at 72 HPD. Only a subset of new branches are indicated with green arrows to avoid obscuring the dendrite. Statistical analysis in I is the same as in G.

Nociceptive Class IV neurons like ddaC have the most complex arbors of the larval sensory neurons. Class I dendritic arborization neurons including ddaE have simpler arbors [35], are proprioceptive [65], and respond to folding of the cuticle during larval crawling [66,67]. ddaC neurons continue to add branches throughout larval life, but ddaE neurons obtain their final shape early in larval life before the time we injure them [11]. ddaE neurons respond to dendrite removal by re-initiating branching and add branches to recapitulate the pre-injury number of branch points [11,42]. If only one dendrite is removed, then the new branches are added to the remaining dendrites and only minimal outgrowth is involved as the new branches are typically very short [11,37,68]. As different types of neurons likely contain different sets of voltage-gated channels, we wished to test whether calcium levels in the cell body would also increase after axon and dendrite injury. Indeed, we found that GCaMP6f fluorescence followed a similar pattern after both types of injury in these cells (Fig 4A–4E). If VGCCs are important for injury signaling and initiation of regeneration, rather than for large scale growth, ddaE neurons might also exhibit a deficit in branch addition after removal of a single dendrite. We compared branching in control, Ca-α1D and stj RNAi neurons 24 hours after removal of the large comb-shaped dendrite. Control neurons added on average almost 6 new branches to the arbor, and this number was reduced in Ca-α1D and stj RNAi neurons (Fig 4F and 4G). To test whether regeneration would recover at later time points as in Class IV neurons, we assayed branch addition 72 hours after removal of the comb dendrite. At this time, similar numbers of branches were added in control, Ca-α1D and stj RNAi neurons (Fig 4H and 4I). This data is consistent with VGCCs promoting early dendrite injury responses across multiple cell types.

### AC8 family member Ac78C promotes dendrite regeneration

**Fig 5:**
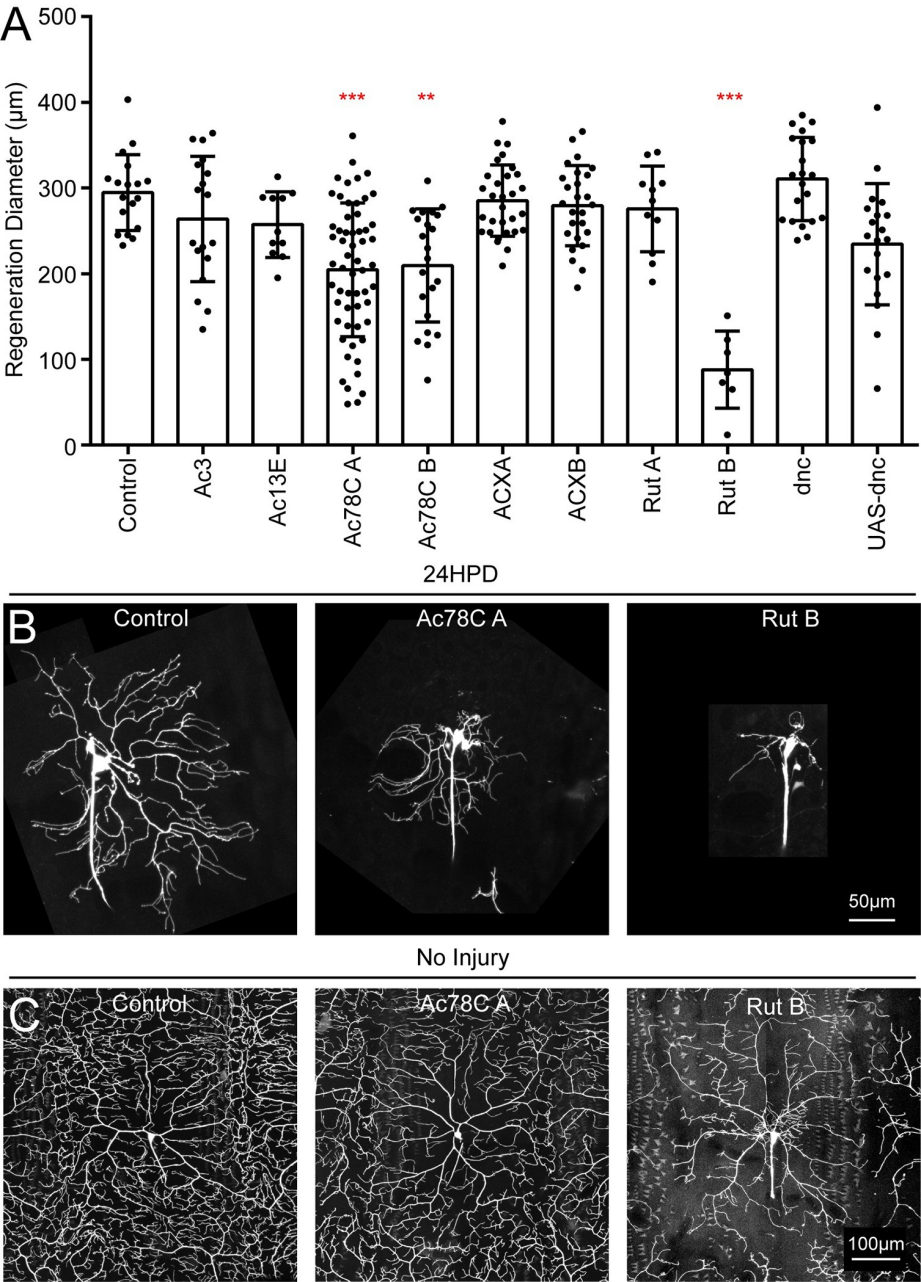
Dendrite regeneration screen with adenylyl cyclase (AC) candidates. (A) Dendrites of ddaC neurons expressing RNAi hairpins to target candidate ACs were severed at time 0. Maximum regeneration diameter was measured 24HPD. (B) Representative images are shown for control, Ac78C A and Rut B RNAi lines. **, p < .01; ***, p < .001 with a Kruskal–Wallis one-way analysis of variance (ANOVA), each condition compared with the control with Dunn’s multiple comparisons test. (C) Morphology of uninjured ddaC neurons is shown for control, Ac78C A and Rut B RNAi lines. Dendrites of Rut B RNAi neurons did not reach the edge of the territory, had sparse coverage and extra branching near the soma. Error bars in (A) are standard deviations.

cAMP promotes axon regeneration, and we therefore wished to determine whether it might also be involved in dendrite regeneration. cAMP is produced by adenylyl cyclases (ACs) and eliminated by phosphodiesterases. To probe broadly whether cAMP levels might influence dendrite regeneration, we knocked down and overexpressed the phosphodiesterase dunce (dnc) and measured regeneration diameter 24h after injury (Fig 5A). Regeneration seemed slightly elevated in the RNAi condition and slightly reduced in the overexpression background, but neither result was significant. We therefore moved on to testing specific ACs. In mammals there are nine classes of transmembrane ACs, which are typically regulated by heterotrimeric G proteins acting downstream of G protein coupled receptors. However, three classes (AC1, AC3 and AC8) can also be activated by calcium [69,70]. The Drosophila genome encodes 13 putative ACs (flybase.org). We prioritized genes expressed in neurons that could be targeted by RNAi transgenic lines available from the Bloomington Drosophila Stock Center (BDSC). All ACs belonging to the mammalian calcium regulated families were included: AC1 (rut), AC3 (Ac3) and AC8 (Ac78C). We also included Ac13E, ACXA and ACXB even though they are not in the calcium regulated families because they are significantly expressed in neurons based on publicly available transcriptome data (flybase.org). Two different RNAi hairpins targeting Ac78C and one hairpin targeting rut reduced dendrite regeneration (Fig 5A and 5B). When performing the rut RNAi injuries with the RNAi line that reduced regeneration, we noticed that the morphology of uninjured neurons was disrupted with more branching close to the cell body and sparse coverage in distal regions of the arbor while morphology of Ac78C RNAi neurons was normal before injury (Fig 5C). Because Ac78C specifically affected dendrite regeneration, we chose to focus our analysis on it rather than rut, which may more broadly affect dendrite growth.

### cAMP is produced by Ac78C after dendrite injury and promotes early dendrite outgrowth

**Fig 6:**
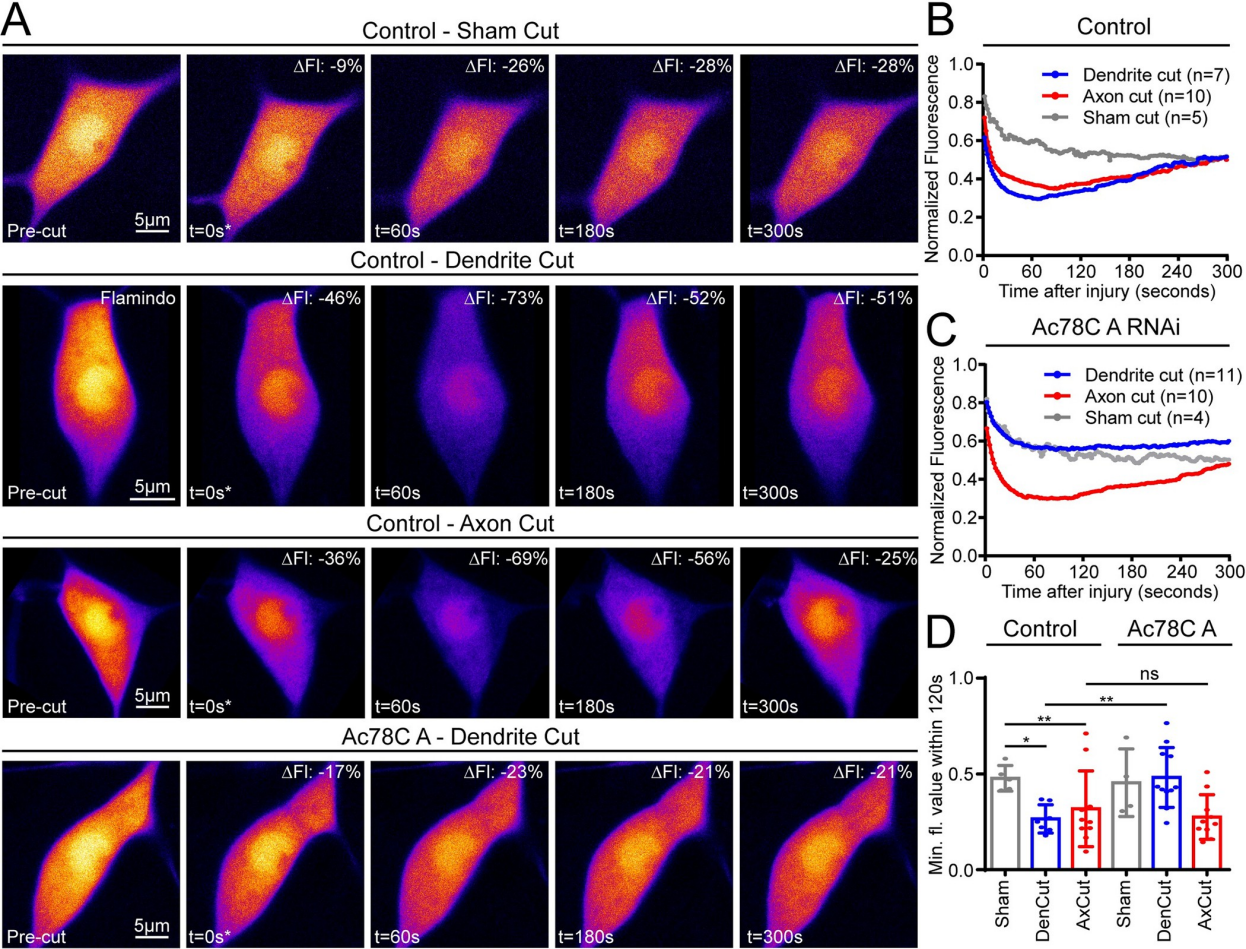
cAMP is produced after axon and dendrite injury and Ac78C is required after dendrite injury. cAMP levels were measured after injury of ddaC neurons for five minutes with the Flamindo2 biosensor. (A) Example images of time points within the 5-minute videos are shown for control sham, dendrite, and axon cuts as well as Ac78C RNAi dendrite cuts. Percent loss of fluorescence is shown for each image in the top right corners. (B) Average fluorescence intensity over time is plotted for axon, dendrite, and sham cut conditions. Note the deep trough and subsequent fluorescence recovery after axon and dendrite cuts (red and blue traces respectively) compared to a sham injury (gray trace). (C) Neurons expressing Ac78C RNAi were tested for dynamic cAMP levels after injury. Note that the dendrite cut condition closely mirrors the sham cut condition, but axon cut response is largely unaltered. (D) Minimum fluorescence values are shown for sham, dendrite, and axon cut conditions, Kruskal Wallis with Dunns multiple comparisons was used to compare control sham to control dendrite cut and control sham to control axon cut. To compare control dendrite cut to Ac78C dendrite cut and control axon cut to Ac78C axon a Mann Whitney test was used. Error bars for (B and C) are omitted for clarity, but relevant error information is contained in (D), which are standard deviations.

To determine whether Ac78C produces cAMP in response to dendrite injury, we first had to characterize cAMP levels after cutting. We therefore generated transgenic Drosophila that express the cAMP sensor Flamindo2 [71] under UAS control. Flamindo2 decreases in fluorescence in response to cAMP. To control for bleaching, we included a sham cut condition. For the sham cut, we positioned the UV pulsed laser near, but not on, a neuron. This exposure caused about 20% initial bleaching of Flamindo2 in the soma, with slower additional bleaching due to confocal imaging (Fig 6A and 6B). In contrast, when we severed an axon or a dendrite with the UV cutting laser there was a 30–40% initial decrease in fluorescence, which continued to decrease until hitting a “peak” about 30–60 seconds after the injury (Fig 6B). While the sham cut condition continued gradual photobleaching, the axon and dendrite cut conditions recovered fluorescence, up to about 50% of pre-cut level by the end of the 5-minute video (Fig 6A and 6B and S4 and S5 Movies). Both the deeper trough, and recovery of fluorescence strongly suggest that cAMP is being produced in class IV neurons on the seconds timescale after both axon and dendrite injury. To determine whether Ac78C was responsible for this early cAMP rise, we monitored Flamindo2 fluorescence in Ac78C RNAi neurons. Ac78C RNAi eliminated the dip and rise of Flamindo2 after dendrite injury (Fig 6A, 6C, and 6D and S6 Movie) indicating that cAMP production after dendrite injury requires Ac78C. The Flamindo signal in response to axon injury was not changed by Ac78C RNAi so we do not know which AC acts after axon injury.

**Fig 7:**
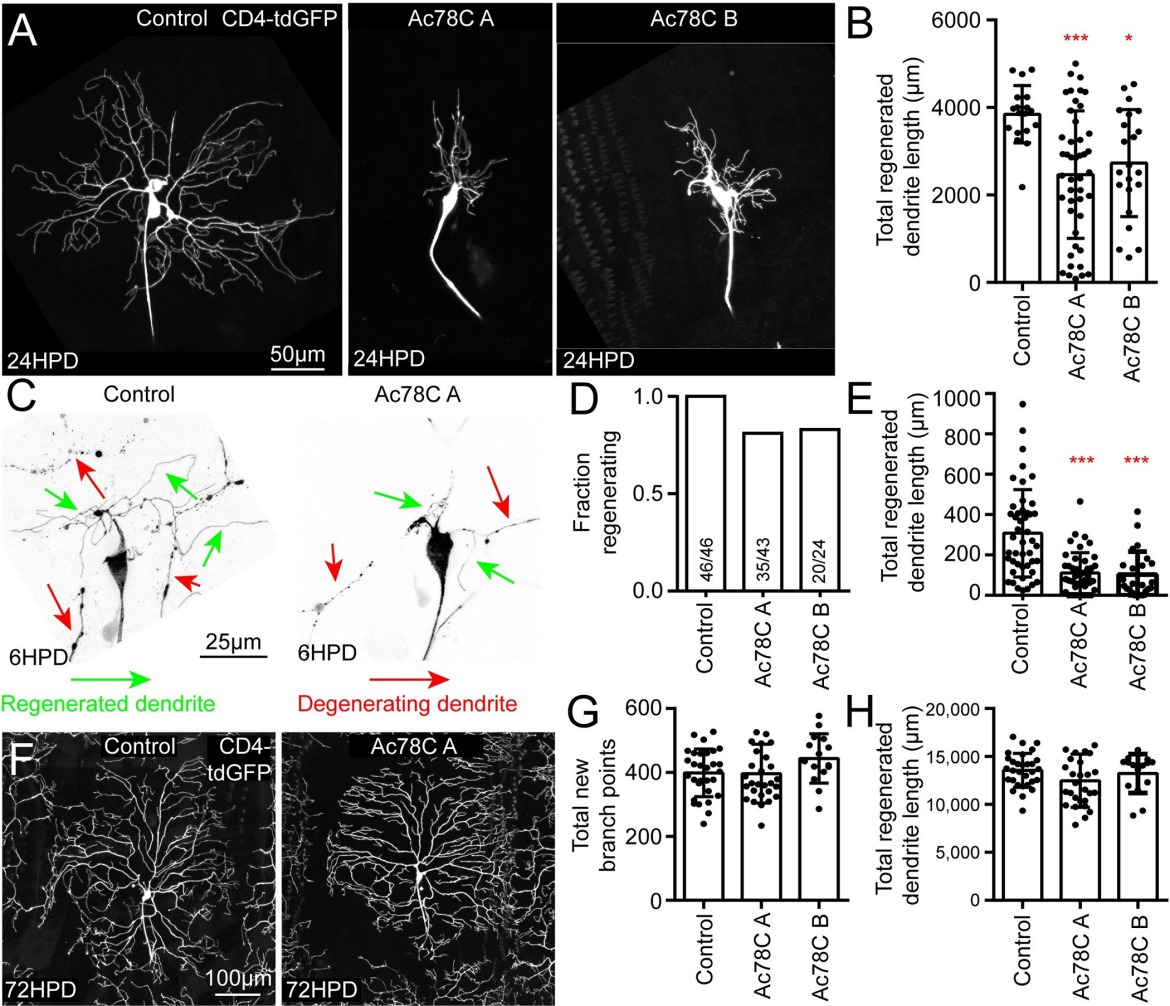
Ac78C is required for early initiation of dendrite regeneration. Dendrite regeneration of ddaC neurons was assayed with two independent Ac78C RNAi hairpins at 6, 24, and 72HPD. Example images for each time point are shown in (C), (A) and (F) respectively. (B) Total regenerated dendrite length was quantified for control and Ac78C RNAi conditions at 24HPD from the same images used to calculate diameter in Fig 6. Fraction regenerating and total regenerated dendrite length were quantified at 6HPD in (D) and (E). Total new branch points and total regenerated dendrite length were quantified at 72HPD in (G) and (H). Note that control data in (B) is the same set as displayed in Fig 2, and control data in (D) through (H) comprises the control data for 6HPD and 72HPD in Fig 3 and an additional set of control data; the additional control data was obtained at the time of the second Ac78C RNAi data (Ac78C B) as it was done later than the other 6h and 72h experiments. Statistics in (D) is a Chi-square test (all ns) and tests in all other graphs are Kruskal-Wallis ANOVAs with Dunn’s multiple comparisons test. *, p<0.05, **, p<0.01, ***, p<0.001. All error bars are standard deviations.

To test whether Ac78C is involved in initiation of dendrite regeneration like VGCCs, we first measured ddaC dendrite regeneration at 24h after arbor removal. The reduction in total dendrite length (Fig 7A and 7B) 24 hours after dendrite removal was similar to that of VGCC RNAi neurons suggesting that Ac78C may also act to promote timely initiation of dendrite regeneration. We therefore assayed dendrite regeneration at 6h and 72h after arbor removal. Like VGCC RNAi, Ac78C RNAi neurons had reduced regeneration 6h after injury (Fig 7C–7E), but normal levels of regeneration 72h after injury Fig 7F–7H). We conclude that cAMP produced by Ac78C promotes timely initiation of dendrite regeneration.

### cAMP is produced downstream of VGCC activation after dendrite injury

**Fig 8:**
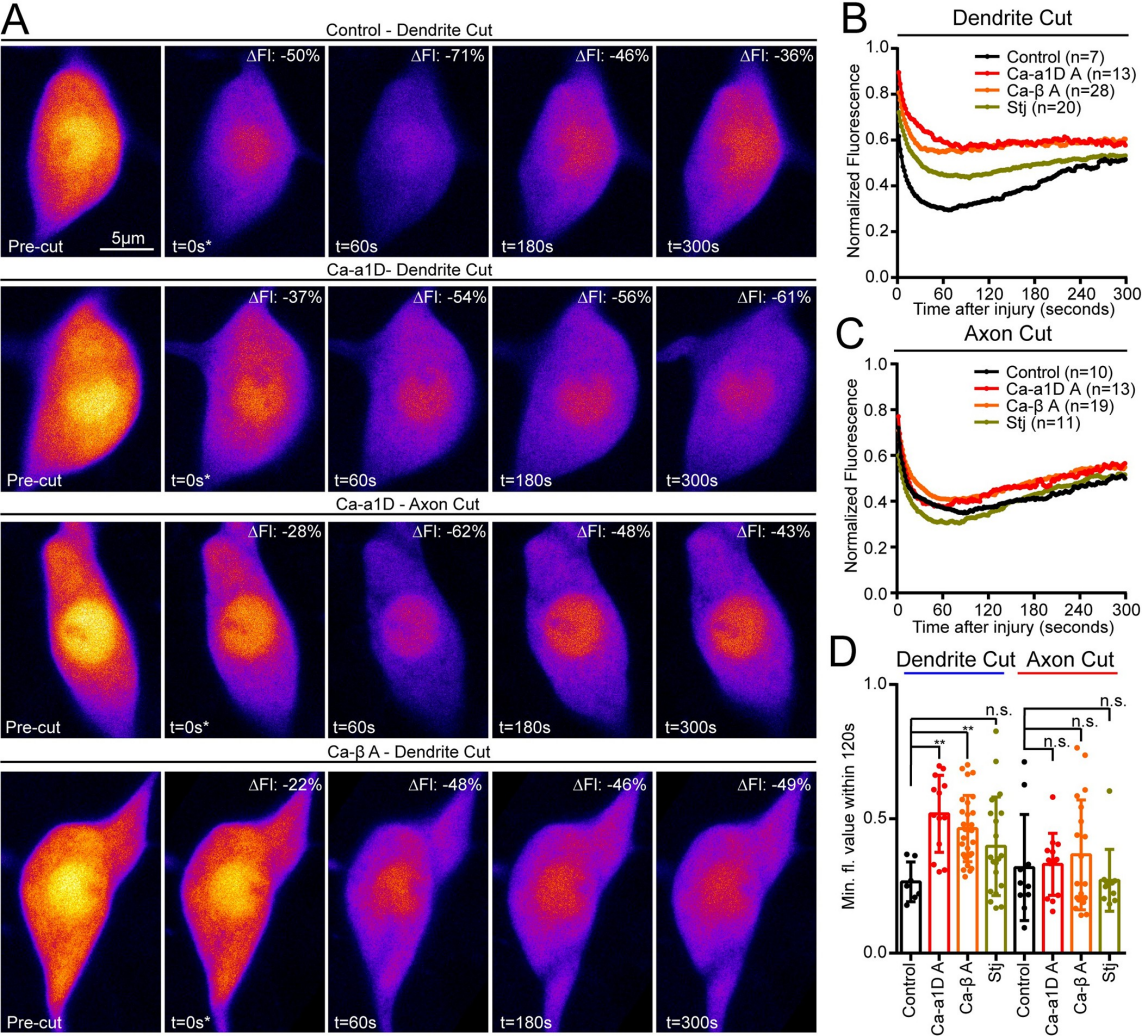
VGCCs are required for cAMP production after dendrite but not axon injury. Flamindo2 fluorescence was monitored in ddaC neurons expressing RNAi hairpins to target VGCC subunits. (A) Example time series for control dendrite cut, and both dendrite and axon cuts with Ca-α1D RNAi, are shown. Average fluorescence intensity over 5 minutes is graphed for dendrite cut in (B) and axon cuts in (C). Note that control dendrite and axon cuts (black traces in (B) and (C) are the same data sets displayed in blue and red, respectively, in Fig 5B and 5C. (D) Minimum fluorescence value is displayed for each genotype and compared to its respective control with Kruskal-Wallis one way analysis of variance (ANOVA), with Dunn’s multiple comparisons test, **, p<0.01. Error bars in (D) are standard deviations. Error bars are omitted in (B) and (C) for clarity, but relevant error information is contained in (D).

It has been suggested that cAMP is the effector of increased calcium after axon injury [2,19], although the AC responsible for cAMP production has not been identified and there is no direct evidence to support this model. We wanted to directly assess whether calcium influx was required for cAMP production after dendrite injury. To this end, we knocked down VGCC subunits Ca-α1D, Stj and Ca-β, and monitored cAMP levels with Flamindo2 after axon and dendrite injury (Fig 8A). We saw no effect on cAMP production after axon injury (Fig 8C), which is not surprising as these RNAi conditions did not block calcium elevation after axon injury. However, cAMP production after dendrite injury was eliminated by Ca-α1D and Ca-β RNAi (Fig 8A, 8B and 8D). Stj knockdown also reduced the decrease in fluorescence after dendrite injury (Fig 8B and 8D) but using only the minimum values did not reach statistical significance (Fig 8D). This data shows that VGCCs are required to activate cAMP production after dendrite injury. We have thus demonstrated that calcium entry is upstream, and essential for, cAMP generation during dendrite injury signaling.

## Discussion

Axon and dendrite injury both result in regeneration, but we previously showed that DLK/JNK/fos signaling is only activated by axon injury, and is neither activated by dendrite injury nor required for dendrite regeneration [11]. Whether other injury signals are shared between axons and dendrites has not been determined. Here we show that both axon and dendrite injury lead to similar calcium and cAMP peaks in the minutes after injury. We show that two VGCCs, Ca_v_1 and Ca_v_3 (Ca-α1D and Ca-α1T, respectively), are required for calcium influx after dendrite injury. We go on to show that these same channels are important for prompt initiation of regeneration in neurons with both simple and complex arbors, suggesting that their key role is in injury signaling rather than outgrowth. In genetic backgrounds where the early calcium influx is reduced regeneration eventually catches up, consistent with a role in early signaling rather than growth; this effect is therefore distinct from previously described roles of calcium in dendrite growth [72]. A recently published study on calcium signaling after dendrite injury in Drosophila sensory neurons identified a similar pattern of calcium elevation but could not pinpoint the VGCCs required for the calcium rise [73]. They did find that Ca-α1D RNAi caused a slight reduction in ddaE dendrite regeneration 72h after injury [73], which is different from our finding that, despite an effect on ddaE regeneration 24h after injury (Fig 4G), regeneration was similar in Ca-α1D and control neurons 72h after dendrite injury (Fig 4I). We are not sure about the cause of this discrepancy; it may be that their slightly different quantitation method was more sensitive to a subtle difference, or that our use of RNAi transgenes targeting different channel subunits for all experiments added additional rigor to our findings.

We identify Ac78C as an adenylyl cyclase important for dendrite regeneration and show that it is responsible for cAMP production in response to dendrite injury. Moreover, we demonstrate that dendrite injury-induced cAMP production is blocked by VGCC RNAi, placing VGCC-mediated calcium entry upstream of cAMP signaling. We propose a model in which dendrite injury results in VGCC opening, which in turn activates Ac78C. cAMP then acts as a major output of the calcium signal and stimulates outgrowth of new dendrites. The fact that Ac78C RNAi strongly reduces cAMP after dendrite injury but does not result in a long-term deficit in regeneration suggests that a parallel dendrite injury signaling pathway may exist.

Calcium has long been linked to axon injury signaling, and several downstream pathways have been delineated. At least one of these pathways relies on PKC and so is cAMP-independent; PKCμ is activated by VGCC and ER-derived calcium and phosphorylates HDAC5 to modify axonal microtubules and facilitate transcriptional reprogramming [18,20]. While the cAMP-regulated kinase PKA has been shown to act upstream of DLK to mediate axon regeneration [30], it has not been shown to depend on injury-induced calcium. It has not been possible to determine the relative importance of cAMP to calcium signaling after axon injury as the injury-activated AC has not been identified. Our data suggests that after dendrite injury cAMP is a key effector of calcium injury signaling as Ac78C RNAi had a similar effect on initiation of dendrite regeneration to VGCC RNAi.

While we identified proteins responsible for the calcium and cAMP signals induced by dendrite injury, we did not find genetic backgrounds that eliminated these signals after axon injury. VGCCs have been shown to be required for axon injury signaling in *C*. *elegans* [19] and mammalian neurons [20]. One possible explanation is that VGCCs are more robustly activated by axon injury and so more complete channel reduction would be required to see an effect. In Drosophila Class IV neurons Ca-α1D and Ca-β both play a role in generating calcium spikes that are associated with regeneration; these occur beginning 24 hours after injury [59] and so are distinct from the early calcium increase examined here. We did not systematically test ACs for a function in axon injury signaling, so we can only say that knockdown of Ac78C is sufficient to eliminate cAMP production after dendrite injury and did not affect cAMP generation after axon injury. It is possible that a different AC is activated by axon injury, or that several may act in parallel.

We found that two VGCCs contribute to the increase in calcium after dendrite injury. Ca-α1D is the alpha subunit of the Ca_v_1 (L-type) channel in Drosophila and Ca-α1T is the alpha subunit of Ca_v_3 (T-type). Ca_v_3 channels are typically activated by smaller voltage deviation from resting membrane potential than Ca_v_1 channels and are rapidly inactivated [48], but we could not clearly separate their function here. While there are multiple alpha subunits in Drosophila, there is only one known beta subunit. While it is thought that beta, and to a lesser extent α2δ, subunits are generally important for trafficking of alpha subunits to the plasma membrane [48], the subunit composition of channels in different cell types in Drosophila is not well-defined. Calcium transients precede pruning in Class IV dendritic arborization neurons, and these are partially reduced in *Ca-α1D* and *cac* (Ca_v_2) mutant clones and more strongly reduced in *Ca-β* mutant clones suggesting Ca-β may function with both of those alpha subunits [58]. In Class IV neurons responding to axon injury, Ca-β and Ca-α1D RNAi had similar effects on calcium transients [59]. Thus Ca-α1D and Ca-β likely function together in Class IV neurons, and it is possible Ca-β also works with Ca-α1T. The α2δ subunit stj is important for cac localization at synapses [74,75]. Stj has also been linked to currents produced by Ca-α1D in larval motor neurons [50], so may complex with Ca-α1D in sensory neurons as well.

After dendrite injury, the calcium influx mediated by VGCCs peaks around 10s. Downstream of calcium entry, Ac78C generates cAMP with highest levels 30-60s after injury. Presumably cAMP then activates a signaling pathway that results in more efficient initiation of outgrowth several hours later. The canonical mediator of cAMP signaling is PKA (protein kinase A). However, several other cAMP effectors including Epac, a guanine nucleotide exchange factor for Rap type small GTPases, and cyclic nucleotide gated channels (CNG) have been identified [76]. We have not identified the cAMP effector for dendrite injury. After axon injury, PKA acts upstream of DLK [30], so if involved in dendrite regeneration, PKA would need to have a different target. It is very intriguing that although axon and dendrite injury result in similar increases in calcium and cAMP, they lead to different transcriptional responses; for example, the AP-1 transcription factor fos acts only after axon injury to increase levels of its downstream target puckered [11,77].

While Ac78C RNAi strongly reduced cAMP, the effect on dendrite regeneration was quite mild: a delay in initiation. Several possibilities could account for this mild outcome. First, dendrite regeneration could incorporate ongoing outgrowth of class IV neurons during larval development. Indeed, a previous study found that regenerating class IV neurons grow a similar total length to developing class IV neurons over a two day time window [42]. Perhaps after a pause, class IV neurons simply resume ongoing growth after dendrite injury and cAMP promotes rapid resumption. Second, additional injury signals could act in parallel to cAMP. While DLK is absolutely required to initiate axon regeneration in larval class IV neurons in Drosophila [11], in zebrafish it acts in parallel with a sister kinase LZK and only elimination of both completely blocks axon regeneration of motor neurons [78] demonstrating proteins can act in parallel to initiate regeneration. Other proteins that promote dendrite regeneration have been identified, but these likely later during growth rather than early injury signaling [13]. For example, cytoskeletal regulation including microtubule nucleation [37,68], microtubule minus end growth [79], and Rac GTPase signaling [32] are critical for effective dendrite regrowth. Similarly, interactions with the extracellular matrix regulate regrowth [80,81], as does cellular metabolism [10] and exocytosis [82]. Determining whether additional signals communicate dendrite injury to the soma in parallel to calcium and cAMP, and help distinguish it from axon injury, remains a major challenge in the nascent field of dendrite regeneration.

## Materials and methods

### Drosophila stocks and rearing

All RNAi lines used in this study were obtained from the Bloomington Drosophila Stock Center or the Vienna Drosophila Resource Center [45]. γTub37c VDRC#25271 is used as a control as it is not expressed in somatic tissues [83]. Transgenic tester lines used in this study include:

UAS-dicer2 (second chromosome); ppk:Gal4, UAS:GCaMP6F/TM6B

Ppk:Gal4, ppk:EGFP, ppk:CD4-tdGFP, UAS:dicer2-nlsBFP/TM3, Tb-RFP

UAS:Flamindo2 (2^nd^ chromosome); ppk:Gal4, UAS:dicer2-nlsBFP (third chromosome)

ppk:Gal4 (2^nd^ chromosome)

Females of tester lines were crossed to males of RNAi lines, or in the case of 17xUAS-ER-GCaMP-210, ppk:Gal4 females were crossed to males of 17xUAS-ER-GCaMP-210. For a complete list of stocks, please see the Reagent table (S2 Table).

Animals were reared on standard Drosophila media either in vials or 35mm petri dishes. They were incubated at 25C for all experiments. For 72h dendrite regeneration assays, dendrites were severed at mid-second instar and were incubated at 25C until imaging 72h later. For all other experiments with larvae mid third instar larvae were used and were kept at 25C before and after injury. For the adult ddaC analysis recently eclosed flies (<24h) were used.

### Generation of UAS-Flamindo2 transgenic Drosophila

The coding sequence of Flamindo2 was PCR amplified from addgene plasmid 73938 (https://www.addgene.org/73938/) and inserted into the pUAST vector [84]. Embryo injections were performed by BestGene (https://www.thebestgene.com/) and insertions were mapped based on eye color.

### Larval mounting and selection of neurons

There are 20 dorsal ddaC neurons in fly larvae, 10 on each side. For all experiments, neurons from within abdominal segments 3 to 5 were used. No more than one neuron per animal was injured/imaged for each experiment. For all experiments, larvae were placed on a microscope slide on top of a small dried agar pad (for immobilization) and had a coverslip taped on top. No anesthesia was used.

### Image acquisition and analysis

The following Zeiss microscopes (Thornwood, NY) were used in this study:

1. LSM800 inverted confocal on an Axio Observer Z1 stand and equipped with GaAsP detectors, a Zeiss Plan-APOCHROMAT 63x DIC (oil, 1.4NA) objective, and a MicroPoint UV pulsed laser (Oxford Instruments);
2. LSM800 upright confocal on an Axio Imager.Z2 stand and equipped with GaAsP detectors and Zeiss Plan-APOCHROMAT 63x DIC (oil, 1.4NA) and Zeiss Plan-APOCHROMAT DIC (UV) VIS-IR 40x (oil, 1.3NA) objectives;
3. Zeiss Axio Imager.M2 widefield equipped with an AxioCam 506 mono camera and Zeiss Plan-APOCHROMAT 63x DIC (oil, 1.4NA), Zeiss EC Plan NEO FLUAR 40x (oil, 1.3NA) objectives and a MicroPoint UV pulsed laser (Oxford Instruments).
4. LSM700 inverted confocal on an Axio Observer Z1 stand with standard detectors and a Zeiss Plan-APOCHROMAT 63x DIC (oil, 1.4NA) objective.

The MicroPoint lasers were used to precisely sever axons or dendrites; microscope #1 was used for Flamindo2 injury experiments and all other injuries were performed with microscope #3. All GCaMP assays were done on scope #3. All Flamindo2 assays were done on scope #1. Adult imaging assay was done on scope #1. Morphology of uninjured neurons was assessed with microscope #2. For all dendrite regeneration assays (class I and class IV, for 6, 24, and 72hr), initial injury was performed with scope #3. For 6hr class IV and 24hr class I dendrite regeneration experiments, image at t = 0 was on scope #3 and t = 24hr on either scope #2 or #4. For 24hr and 72hr class IV regeneration experiments, no image was acquired at t = 0 and the image at t = 24/72hr was acquired on scope #2. All images were analyzed with ImageJ/Fiji (https://fiji.sc/).

### GCaMP6f imaging and quantitation

A MicroPoint UV pulsed laser (Oxford Instruments) mounted to a Zeiss AxioImager.M2 widefield scope was used to sever axons or dendrites. The crosshairs were positioned over the region of interest (ROI) on a neuron and image acquisition was started. The default GFP filter was rotated out and a dichroic mirror was rotated in as the image was started. After filter switching (1-2s after initiation of image acquisition), the UV laser was used to sever the ROI as the image series was being acquired.

For the experiments presented in Figs 1, 4, and S2–S4, images were acquired for 2 minutes after injury; for experiments presented in Figs 2 and part of S6, a different dichroic mirror was used and images were acquired for 1 minute after injury.

For quantification of GCaMP6f intensity, a polygon was drawn around the cell body and fluorescence intensity was measured over time with the Time Series Analyzer plugin in ImageJ. Images were acquired at a rate of 3 frames per second (FPS). All images in movies for which t<0 were normalized to a pre-cut image (i.e. image 1 fluorescence intensity = 1 and all subsequent images are normalized to 1). “Max normalized fluorescence” shown in Figs 1, 2, 4, and S4 are the highest average fluorescence intensity of the whole cell body at any frame per video, representing the “peak brightness” of the whole cell body. Class I and Class IV experiments were performed and quantified identically. Raw measurements can be found in the Data table (S1 Table). Because the dichroic mirror that allows laser cutting and imaging simultaneously was changed during this project, absolute and relative intensities are different between experiments in Figs 2 and S6 and all other GCaMP experiments presented here.

### Class IV (ddaC) dendrite regeneration assay

This assay has been well described in our recent work [33,68,82]. Briefly, class IV dendrites are severed at the base, 3–5 microns from the soma. Larvae are recovered from the microscope slide and kept in a small piece of fly media for 24 hours, when the same neuron is imaged again. We quantify regeneration diameter (longest line one can draw across regenerated arbor), total regeneration length (sum of all neurites traced in Simple Neurite Tracer (SNT) plugin in FIJI), and total branch points (also generated by SNT). All neurons were traced manually in the SNT plugin [85]. The 6-hour and 72-hour dendrite regeneration assays were performed identically, except neurons are imaged at 6 or 72HPD rather than 24HPD. SNT was used to trace arbors.

### Class I (ddaE) dendrite regeneration assay

We severed the dorsal comb-shaped dendrite of class I neurons before the first branch point and took an image pre-cut. After removal of this dendrite, other dendrites add new branch points [11] and we took another image at 24HPD and quantified the number of new branch points at 24HPD.

### Uninjured ddaC dendrite morphology

For Fig 5, baseline ddaC morphology was captured by mounting a 3^rd^ instar larvae slightly tilted to one side (as distal dendrites are located more laterally and can be hard to acquire with larvae mounted straight and upright), and acquiring a tiled image large enough to capture the full neuron and all its dendrites on scope #2.

### Flamindo2 cAMP assay

The microscope used for these experiments was the LSM800 inverted confocal. Since simultaneous UV laser cutting and imaging is not possible on this setup, we took a close-up image of the neuron before any injury. We then used a MicroPoint UV laser to sever dendrites or axons before switching the light path from eyepieces to camera as quickly as possible. Because of this limitation, the image acquisition was started 3–6 seconds after the injury. For sham cuts, the UV laser was aimed within a few microns of the cell body but not on any axonal or dendritic process, as a control for bleaching from the UV cutting laser that happens during injury.

After dendrite, axon, or sham cut, we imaged the neuron for 5 minutes at a frame rate of about 0.55FPS (172 frames in 310 seconds). Because the frame rate was so slow, keeping the soma perfectly in focus was extremely difficult. The extra 10 seconds was built into the acquisition settings to have 6 “free” frames–if the neuron went out of focus for a frame, that frame could be deleted to improve the quality of the quantification. Thus, all videos have up to 6 out-of-focus frames deleted to maintain a total video time of 5 minutes.

For quantification, the fluorescence intensity of the soma in each post-injury image was normalized to the fluorescence intensity of its respective pre-injury image. To better help with comparing traces of data from a biosensor that goes down in fluorescence upon substrate binding (as opposed to going up, like GCaMP), we quantified the “minimum fluorescence value” to be an analog of the “peak” of a GCaMP trace. This quantification is the lowest average fluorescence intensity of the cell body within the first 120 seconds of video (as “peaks” seem to be somewhere in the 30–90 second range).

### Statistics, graphs and figures

All statistics and graph preparation were done in GraphPad Prism. Statistical tests are described in figure legends. *, p < 0.05, **, p < 0.01, ***, p = 0001. Error bars in all figures are standard deviations. For GCaMP6f and Flamindo2 traces, error bars are omitted for clarity of visualization. Figures were prepared in Adobe Illustrator.

## Supporting information

S1 Fig Examples of GCaMP6f signal at early timepoints after axon and dendrite injury. Example frames from movies of GCaMP6f fluorescence in control neurons. These examples are from the set of movies used for the quantitation in Fig 1. GCaMP6f was expressed in Class IV neurons and images were acquired at a rate of 3 per second using a widefield microscope equipped with a pulsed UV laser for severing. Some regions are out of focus because this is a single focal plane from a widefield microscope. These examples show that the timing of how the calcium spreads through the cell has some variability and often combines fairly global increases with a wave that spreads across the cell body from the injury site. The top two examples show dendrite injury and the lower two show axon injury.
(TIF)

S2 Fig “Soft” cuts of axons or dendrites do not saturate GCaMP6f or the microscope detectors. “Explosion” cuts cause a destructive cavitation bubble (A, top, red dotted circle), while the “soft” cuts we use for injury throughout the manuscript are capable of severing neurites without such destruction (A, bottom). Explosion cuts cause a significantly higher signal in ddaC neurons expressing GCaMP6f than “soft” cuts: graphed is the maximum fluorescence value of the brightest pixel in the soma for each condition (B). Sham cuts in which the laser was positioned adjacent to the dendrite were also included. The sham soft cut condition did not result in calcium elevation in ddaC indicating that the calcium signal in axon and dendrite cut conditions is due to direct damage of the neuron. ***, p < .001 with a Kruskal–Wallis one-way analysis of variance (ANOVA), each condition compared with the control with Dunn’s multiple comparisons test. Error bars are standard deviations. Note that standard axon and dendrite cut fluorescence of GCaMP6f is below levels observed with explosion cut and so is not saturated.
(TIF)

S3 Fig Neuronal calcium response to multiple cuts and cuts of varying distances from the soma. Example images of ddaC neurons expressing GCaMP6f after cutting a single dendrite or all dendrites (full cut) are shown in (A). Cut dendrites are signified by red dashed line and, in the single cut condition, intact dendrites are marked by a green dashed line. Cut sites are indicated with red arrow heads. Single dendrite cuts and full dendrite cuts produced very similar GCaMP6f responses, though offset based on time taken to perform cutting (B) (see methods). (C) Schematic of proximal (such as cuts shown in (A) and all other figures), medium, and distal cuts of both the dendrite arbor and axon. Average GCaMP6f traces of these cuts of varying distances is shown in (D) for dendrites and (E) for axons. Note that access to the distal axon is limited past 40–50μm, and as such a distal axon cut is not as far as a distal dendrite cut. Error bars are omitted for clarity.
(TIF)

S4 Fig The calcium spike after neuronal injury and dendrite regeneration are not suppressed by ER calcium channel RNAi. We knocked down RyR, IP3R, Stim, and Orai using RNAi hairpins expressed specifically in Class IV neurons (see Reagent table (S2 Table) for specific tester line and RNAi lines) and assessed calcium influx during neuronal injury with GCamP6f in ddaC neurons. Average GCaMP6f normalized fluorescence intensity in the soma during the 2 minutes after dendrite (A) or axon (B) injury is shown, with peak fluorescence values plotted in (C). (D and E) Dendrite regeneration was assayed 24 hours after all dendrites were removed (24 HPD). Note that control dendrite regeneration data in (C) is from the same set as displayed in Fig 2. ddaC neurons expressed cell shape markers as well as dicer2 and RNAi hairpins targeting a control gene not expressed in somatic cells (γTub37C) or hairpins targeting the indicated proteins. For each arbor the maximum diameter was measured and this is plotted in D. No significant effects on dendrite regeneration were found using Kruskal–Wallis one-way analysis of variance (ANOVA). Each experimental genotype was compared with the respective control with Dunn’s multiple comparisons test. Error bars are omitted in (A) and (B) for clarity, but relevant error information is contained in (C), which are standard deviations. Error bars in (D) are standard deviations.
(TIF)

S5 Fig Lumenal ER-GCaMP shows no meaningful change during axon or dendrite injury. 17xUAS-ER-GCaMP-210 localizes to the inside of the endoplasmic reticulum. We performed cuts with the UV laser on axons, dendrites, and directly on the soma of ddaC neurons expressing ER-GCaMP-210. (A) Axon and dendrite cuts (blue and red trace) did not show any appreciable decrease in fluorescence below uncut control (black trace). In contrast, soma cut caused a rapid decrease in fluorescence (green trace) indicating calcium efflux from the ER lumen. (B) The average minimum fluorescence value within the first 10 seconds for videos in each condition is plotted. No cut, Dendrite Cut, and Axon Cut are all significantly higher than Soma Cut with a Mann-Whitney test. Example time series images of soma cut (C) and dendrite cut (D). % of fluorescence lost compared to pre-cut image over the first 10 seconds of imaging is shown in top right of each frame. Error bars for (A) are not included for clarity, though relevant error information is shown in (B). Error bars in (B) are standard deviations.
(TIF)

S6 Fig Categorization of initial GCaMP6f increase in control and VGCC RNAi neurons. (A) Example images of the timeline of calcium increase in neurons expressing RNAi against Ca-a1T and Ca-a1D B. Top: example of a “wave increase”, note how the upper part of the soma gets brighter first. Middle: example of “global increase”, note how the soma increases in brightness uniformly. Bottom: example of “no increase”, as soma does not get brighter after cut, though calcium increase is visible in the severed portion of dendrite (right side). (B) Proportion of videos that display each of these three categories of fluorescence increase in class IV neurons expressing control and VGCC RNAi hairpins, for both dendrite and axon cut conditions. (C) Comparison of the type of GCaMP6f increases in class I and class IV neurons for both dendrite and axon cut conditions. The data set for Class IV neurons in (C) is a different data set than that in (B). These were collected at different times with different microscope hardware (dichroic mirror); the set in (C) was taken with the same hardware as the Class I data in (C).
(TIF)

S7 Fig Dendrite degeneration is not strongly associated with failure to initiate regeneration 6h after dendrite removal. Example images of control neurons 6 hours after dendrite arbor removal (HPD) are shown (A-C) to demonstrate different degeneration scores: (A) score 0—no trace of degenerating dendrites, (B) score 1- degenerating dendrites still observed but without continuous regions, and (C) score 2 -degenerating dendrites observed with continuous regions present. (D) Example neurons expressing the Wallerian degeneration slow (Wlds) protein are shown at 6 HPD. Note that some newly grown dendrites seem to overlap the degenerating dendrites. Degeneration scores for all genotypes assayed at 6 HPD are summarized in (E). Numbers on the bars are numbers of cells analyzed for each condition. Total regeneration length and total regenerated branch points 6HPD are quantified in (F) and (G) and both are compared with a Mann-Whitney test. Control data is the same set shown in Fig 7D and 7E. No significant difference was found between control and Wlds OE. Error bars are standard deviations.
(TIF)

S8 Fig Adult v’ada neurons do not display obvious morphology deficits with VGCC and AC RNAi. Shown are example overviews of uninjured v’ada neurons in abdomens of adult Drosophila expressing control (A), Ca-α1D (B), Ca-β (C), and Ac78C (D) RNAi. White square denotes area magnified in second row of images. No obvious defects in tiling or complexity are observed.
(TIF)

S1 Table Source data for all graphs. The data table includes source numbers for all main figures and supplemental figures.
(XLSX)

S2 Table Reagent Table. Drosophila RNAi, overexpression, tester, and source lines are listed.
(XLSX)

S1 Methods Methods used in Supplemental Figures. This file includes a description of the additional methods used to acquire data presented in the Supplemental Figures.
(DOCX)

S1 Movie GCaMP6f fluorescence in the cell body of ddaC during the dendrite injury response. Representative video of GCaMP6f during dendrite injury. The cut was on the dendrite at left out of frame. This movie is one of the ones that was quantitated for Fig 1.
(MP4)

S2 Movie GCaMP6f fluorescence in the cell body of ddaC during the axon injury response. Representative video of GCaMP6f during axon injury. The axon is towards the bottom of the movie. This movie is part of the data set quantitated for Fig 1.
(MP4)

S3 Movie GCaMP6f fluorescence in the cell body of a ddaC neuron expressing Ca-α1D A RNAi during dendrite injury response. Representative video of GCaMP6f fluorescence in the cell body after dendrite injury. The dendrite on the right side was severed. The neuron expressed Ca-a1D A RNAi, and this is part of the data set for Fig 3.
(MP4)

S4 Movie Flamindo2 fluorescence in a ddaC neuron after dendrite injury. Representative video of the cAMP biosensor Flamindo2 in the soma of a ddaC neuron after dendrite injury. This reporter fluoresces less as cAMP increases. This movie is part of the data set quantitated in Fig 6.
(MP4)

S5 Movie Flamindo2 fluorescence in a ddaC neuron after axon injury. Representative video of cAMP biosensor Flamindo2 in the soma of a ddaC neuron after axon injury. This movie is part of the data set quantitated in Fig 6.
(MP4)

S6 Movie Flamindo2 fluorescence in a ddaC neuron expressing Ac78C RNAi after dendrite injury. Representative video cAMP biosensor Flamindo2 in the soma of a ddaC neuron after dendrite injury. The neuron expressed Ac78C RNAi hairpins, and is part of the data set quantitated in Fig 6.
(MP4)
