# Supplementary figures and images for: Voltage-gated calcium channels act upstream of adenylyl cyclase Ac78C to promote timely initiation of dendrite regeneration
Source: PLoS Genet. 2024 Aug 26;20(8):e1011388. doi: 10.1371/journal.pgen.1011388 (PMC11379402; doi:10.1371/journal.pgen.1011388)

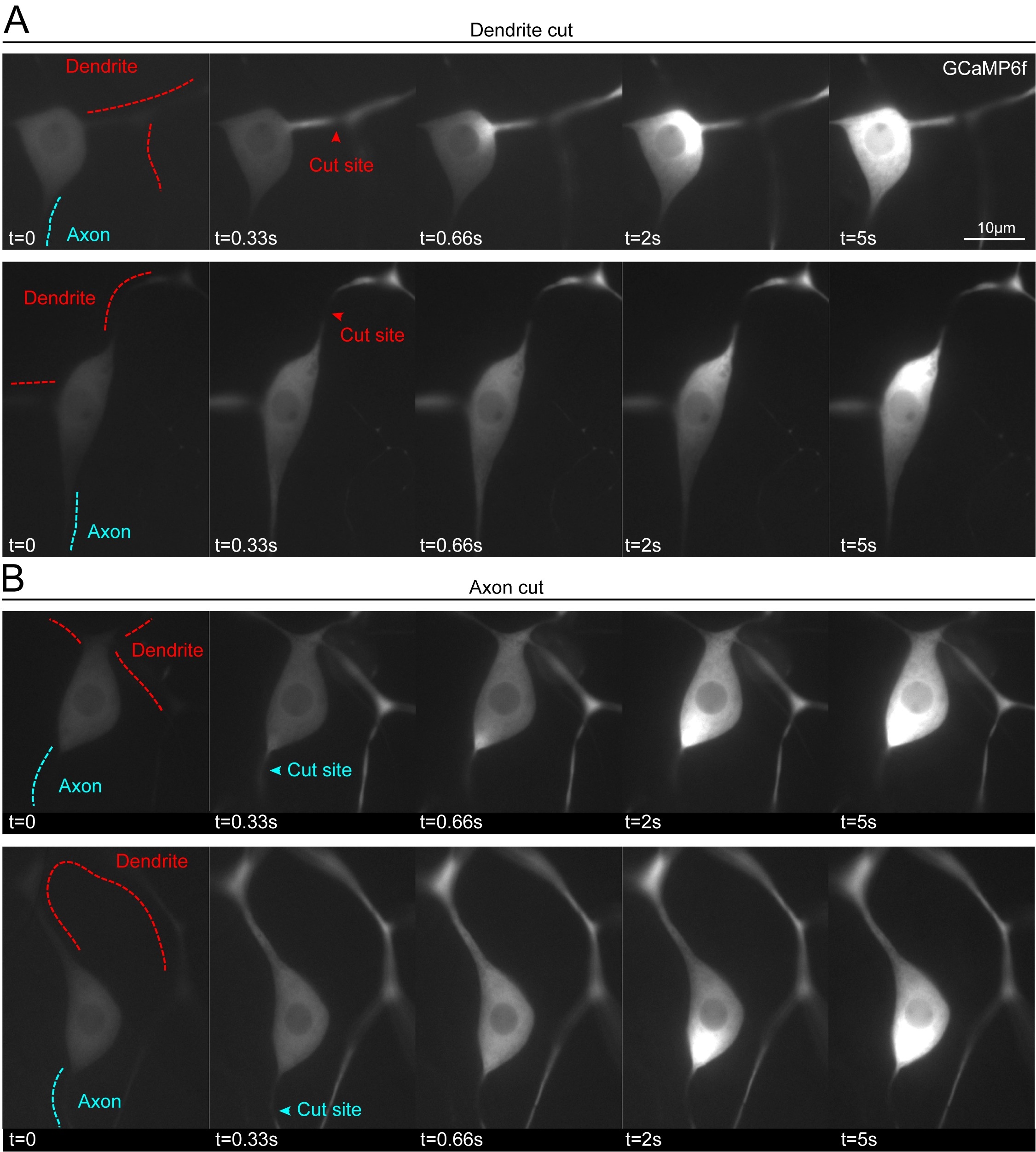

Supplement: S1 Fig — Example frames from movies of GCaMP6f fluorescence in control neurons. These examples are from the set of movies used for the quantitation in Fig 1. GCaMP6f was expressed in Class IV neurons and images were acquired at a rate of 3 per second using a widefield microscope equipped with a pulsed UV laser for severing. Some regions are out of focus because this is a single focal plane from a widefield microscope. These examples show that the timing of how the calcium spreads through the cell has some variability and often combines fairly global increases with a wave that spreads across the cell body from the injury site. The top two examples show dendrite injury and the lower two show axon injury. (TIF) [file pgen.1011388.s001.tif]

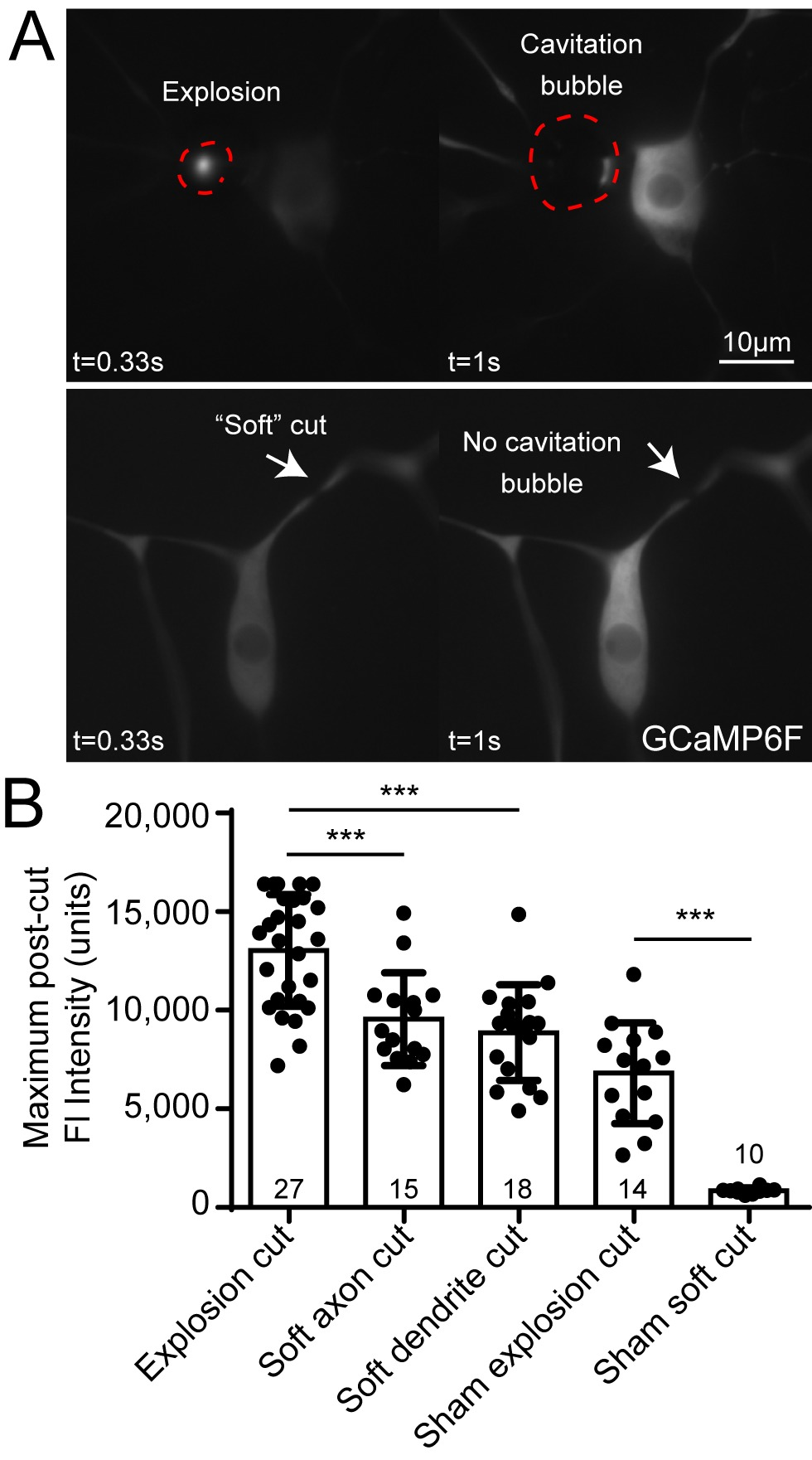

Supplement: S2 Fig — “Explosion” cuts cause a destructive cavitation bubble (A, top, red dotted circle), while the “soft” cuts we use for injury throughout the manuscript are capable of severing neurites without such destruction (A, bottom). Explosion cuts cause a significantly higher signal in ddaC neurons expressing GCaMP6f than “soft” cuts: graphed is the maximum fluorescence value of the brightest pixel in the soma for each condition (B). Sham cuts in which the laser was positioned adjacent to the dendrite were also included. The sham soft cut condition did not result in calcium elevation in ddaC indicating that the calcium signal in axon and dendrite cut conditions is due to direct damage of the neuron. ***, p < .001 with a Kruskal–Wallis one-way analysis of variance (ANOVA), each condition compared with the control with Dunn’s multiple comparisons test. Error bars are standard deviations. Note that standard axon and dendrite cut fluorescence of GCaMP6f is below levels observed with explosion cut and so is not saturated. (TIF) [file pgen.1011388.s002.tif]

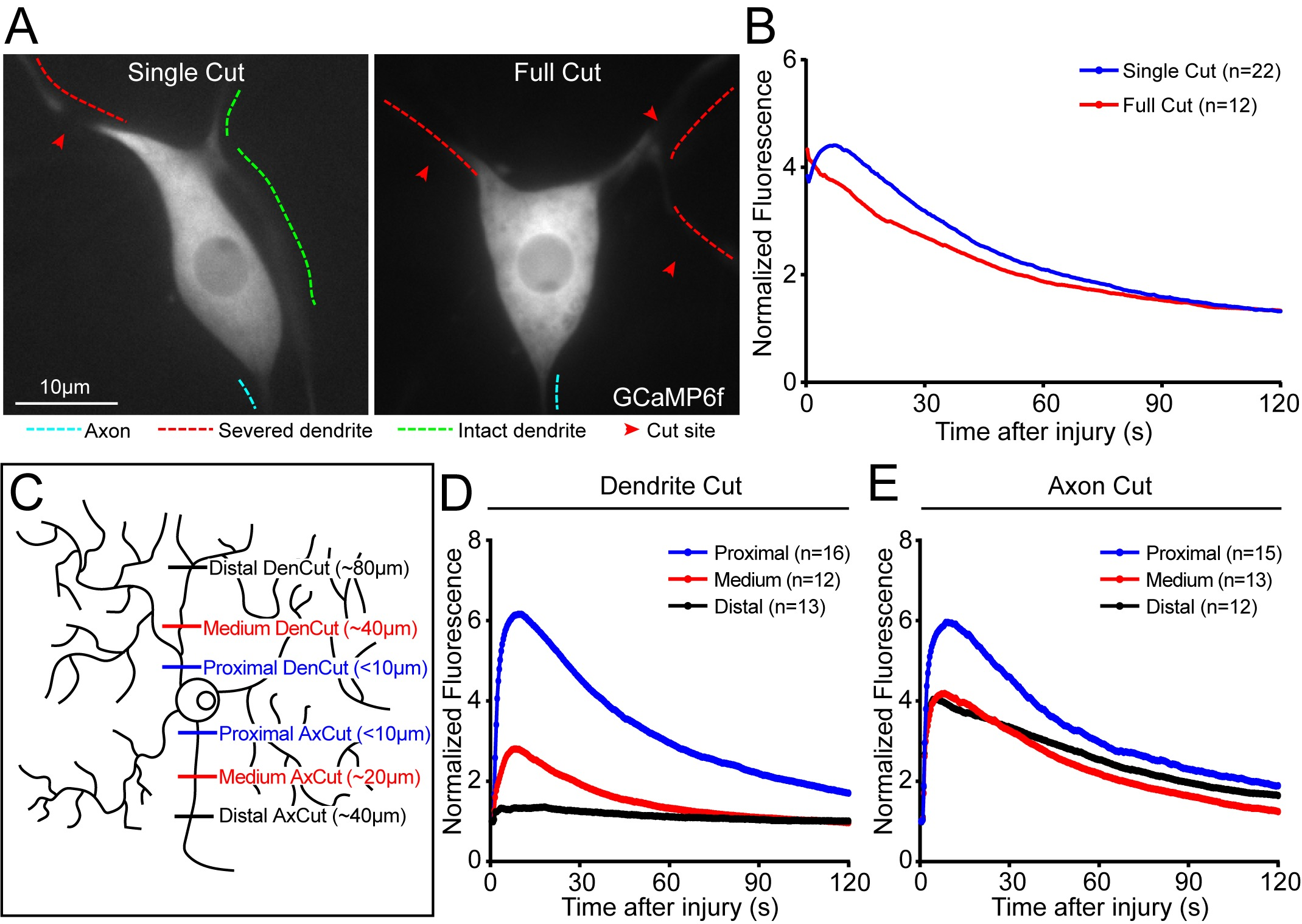

Supplement: S3 Fig — Example images of ddaC neurons expressing GCaMP6f after cutting a single dendrite or all dendrites (full cut) are shown in (A). Cut dendrites are signified by red dashed line and, in the single cut condition, intact dendrites are marked by a green dashed line. Cut sites are indicated with red arrow heads. Single dendrite cuts and full dendrite cuts produced very similar GCaMP6f responses, though offset based on time taken to perform cutting (B) (see methods). (C) Schematic of proximal (such as cuts shown in (A) and all other figures), medium, and distal cuts of both the dendrite arbor and axon. Average GCaMP6f traces of these cuts of varying distances is shown in (D) for dendrites and (E) for axons. Note that access to the distal axon is limited past 40–50μm, and as such a distal axon cut is not as far as a distal dendrite cut. Error bars are omitted for clarity. (TIF) [file pgen.1011388.s003.tif]

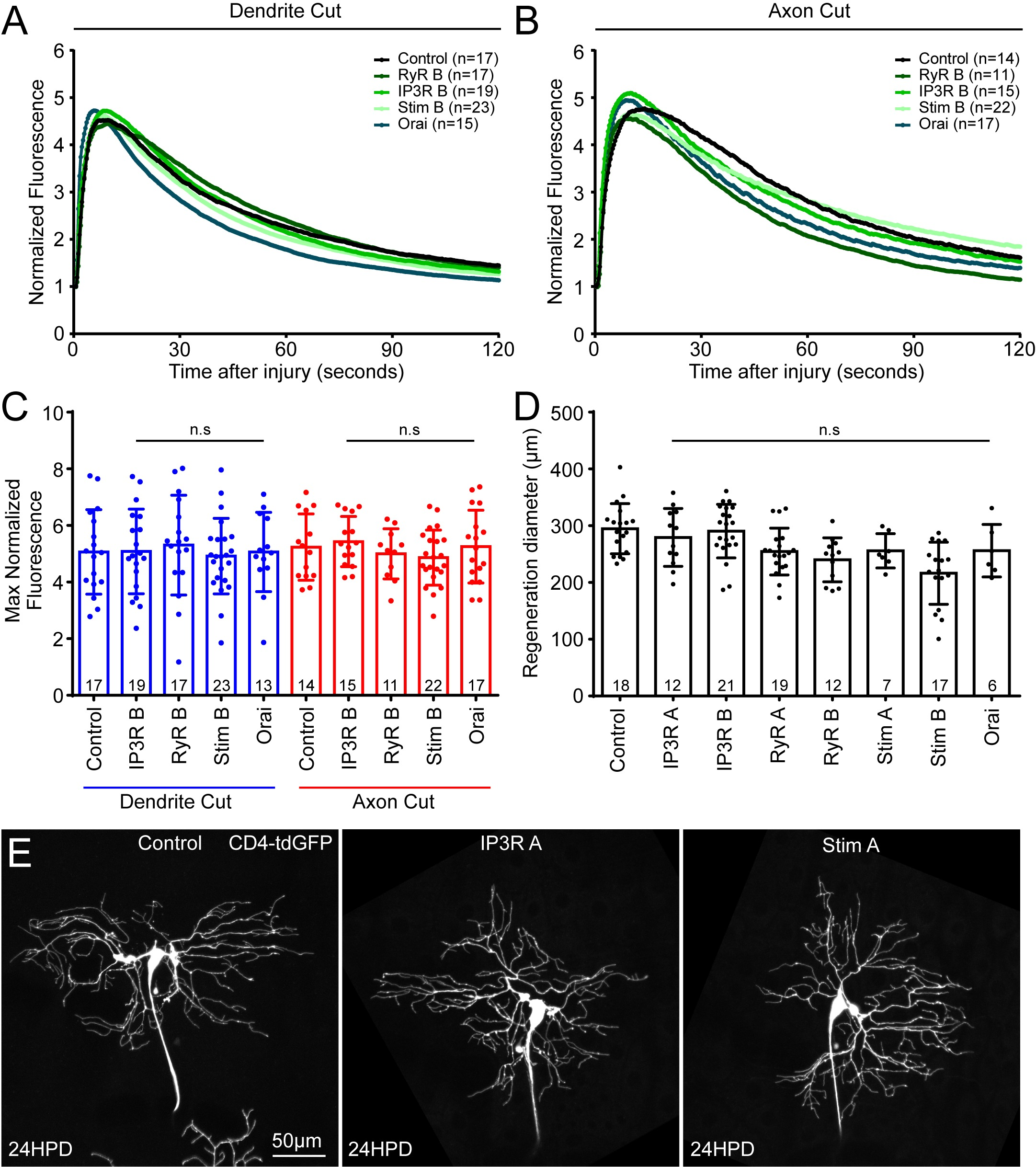

Supplement: S4 Fig — We knocked down RyR, IP3R, Stim, and Orai using RNAi hairpins expressed specifically in Class IV neurons (see Reagent table (S2 Table) for specific tester line and RNAi lines) and assessed calcium influx during neuronal injury with GCamP6f in ddaC neurons. Average GCaMP6f normalized fluorescence intensity in the soma during the 2 minutes after dendrite (A) or axon (B) injury is shown, with peak fluorescence values plotted in (C). (D and E) Dendrite regeneration was assayed 24 hours after all dendrites were removed (24 HPD). Note that control dendrite regeneration data in (C) is from the same set as displayed in Fig 2. ddaC neurons expressed cell shape markers as well as dicer2 and RNAi hairpins targeting a control gene not expressed in somatic cells (γTub37C) or hairpins targeting the indicated proteins. For each arbor the maximum diameter was measured and this is plotted in D. No significant effects on dendrite regeneration were found using Kruskal–Wallis one-way analysis of variance (ANOVA). Each experimental genotype was compared with the respective control with Dunn’s multiple comparisons test. Error bars are omitted in (A) and (B) for clarity, but relevant error information is contained in (C), which are standard deviations. Error bars in (D) are standard deviations. (TIF) [file pgen.1011388.s004.tif]

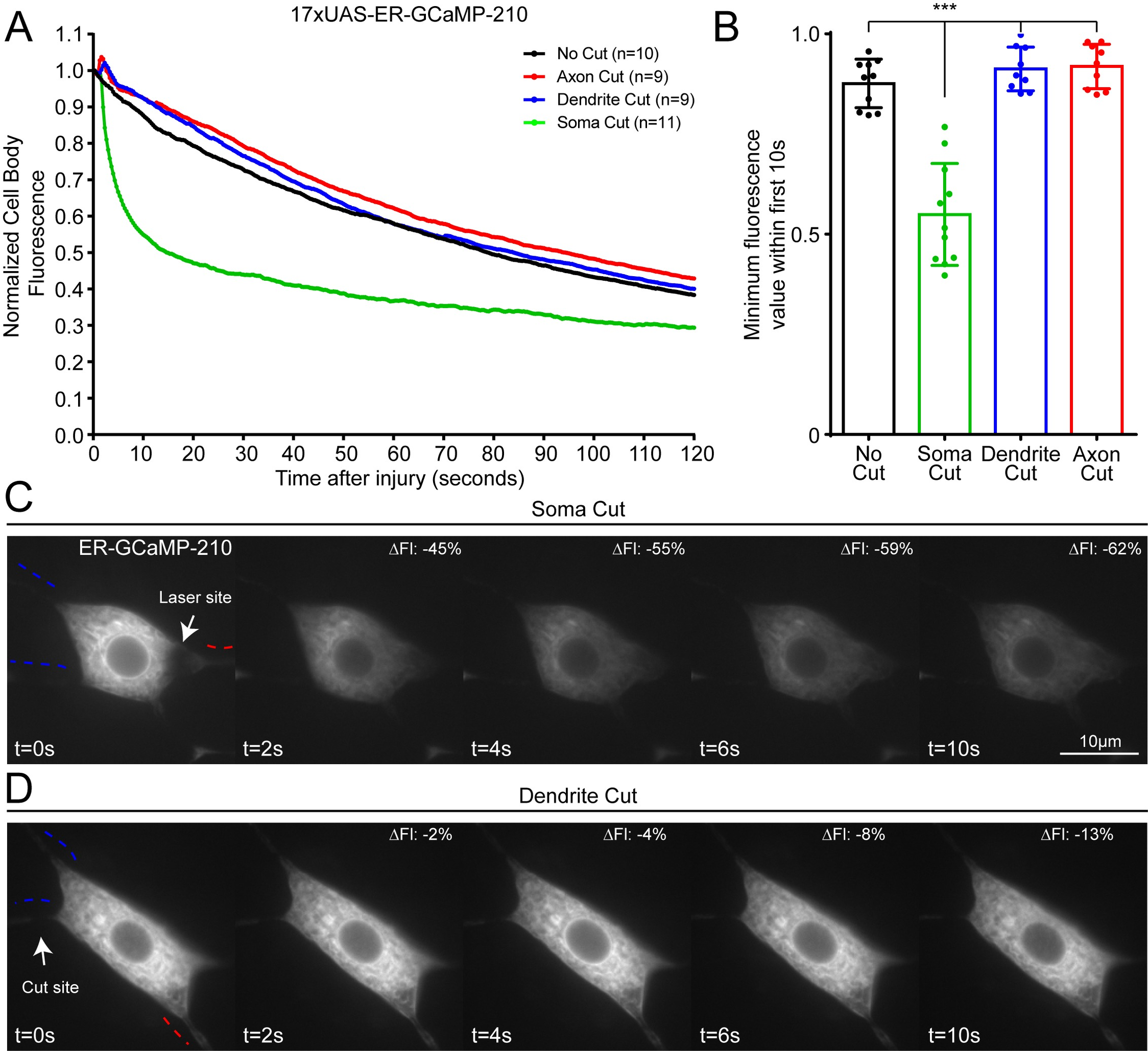

Supplement: S5 Fig — 17xUAS-ER-GCaMP-210 localizes to the inside of the endoplasmic reticulum. We performed cuts with the UV laser on axons, dendrites, and directly on the soma of ddaC neurons expressing ER-GCaMP-210. (A) Axon and dendrite cuts (blue and red trace) did not show any appreciable decrease in fluorescence below uncut control (black trace). In contrast, soma cut caused a rapid decrease in fluorescence (green trace) indicating calcium efflux from the ER lumen. (B) The average minimum fluorescence value within the first 10 seconds for videos in each condition is plotted. No cut, Dendrite Cut, and Axon Cut are all significantly higher than Soma Cut with a Mann-Whitney test. Example time series images of soma cut (C) and dendrite cut (D). % of fluorescence lost compared to pre-cut image over the first 10 seconds of imaging is shown in top right of each frame. Error bars for (A) are not included for clarity, though relevant error information is shown in (B). Error bars in (B) are standard deviations. (TIF) [file pgen.1011388.s005.tif]

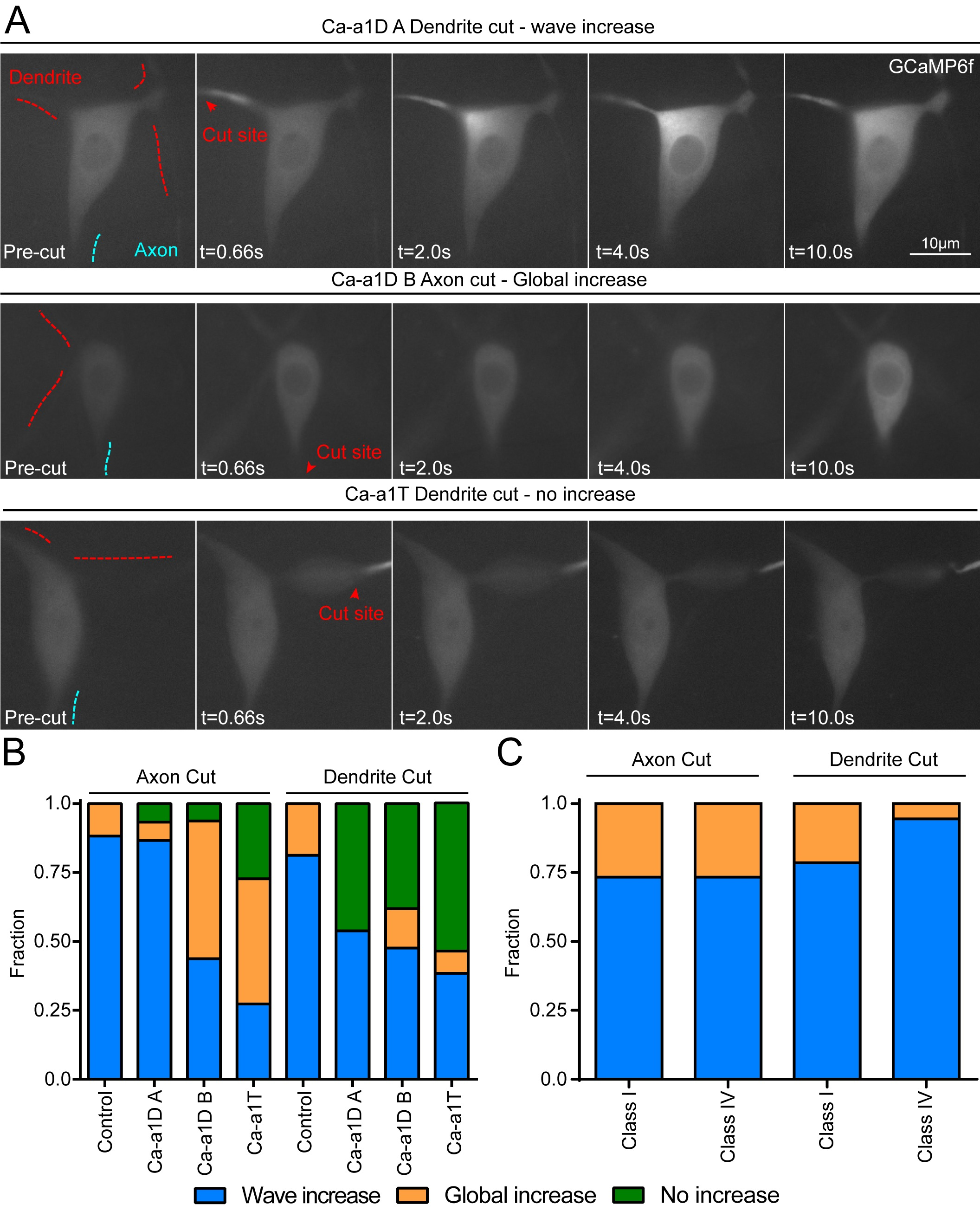

Supplement: S6 Fig — (A) Example images of the timeline of calcium increase in neurons expressing RNAi against Ca-a1T and Ca-a1D B. Top: example of a “wave increase”, note how the upper part of the soma gets brighter first. Middle: example of “global increase”, note how the soma increases in brightness uniformly. Bottom: example of “no increase”, as soma does not get brighter after cut, though calcium increase is visible in the severed portion of dendrite (right side). (B) Proportion of videos that display each of these three categories of fluorescence increase in class IV neurons expressing control and VGCC RNAi hairpins, for both dendrite and axon cut conditions. (C) Comparison of the type of GCaMP6f increases in class I and class IV neurons for both dendrite and axon cut conditions. The data set for Class IV neurons in (C) is a different data set than that in (B). These were collected at different times with different microscope hardware (dichroic mirror); the set in (C) was taken with the same hardware as the Class I data in (C). (TIF) [file pgen.1011388.s006.tif]

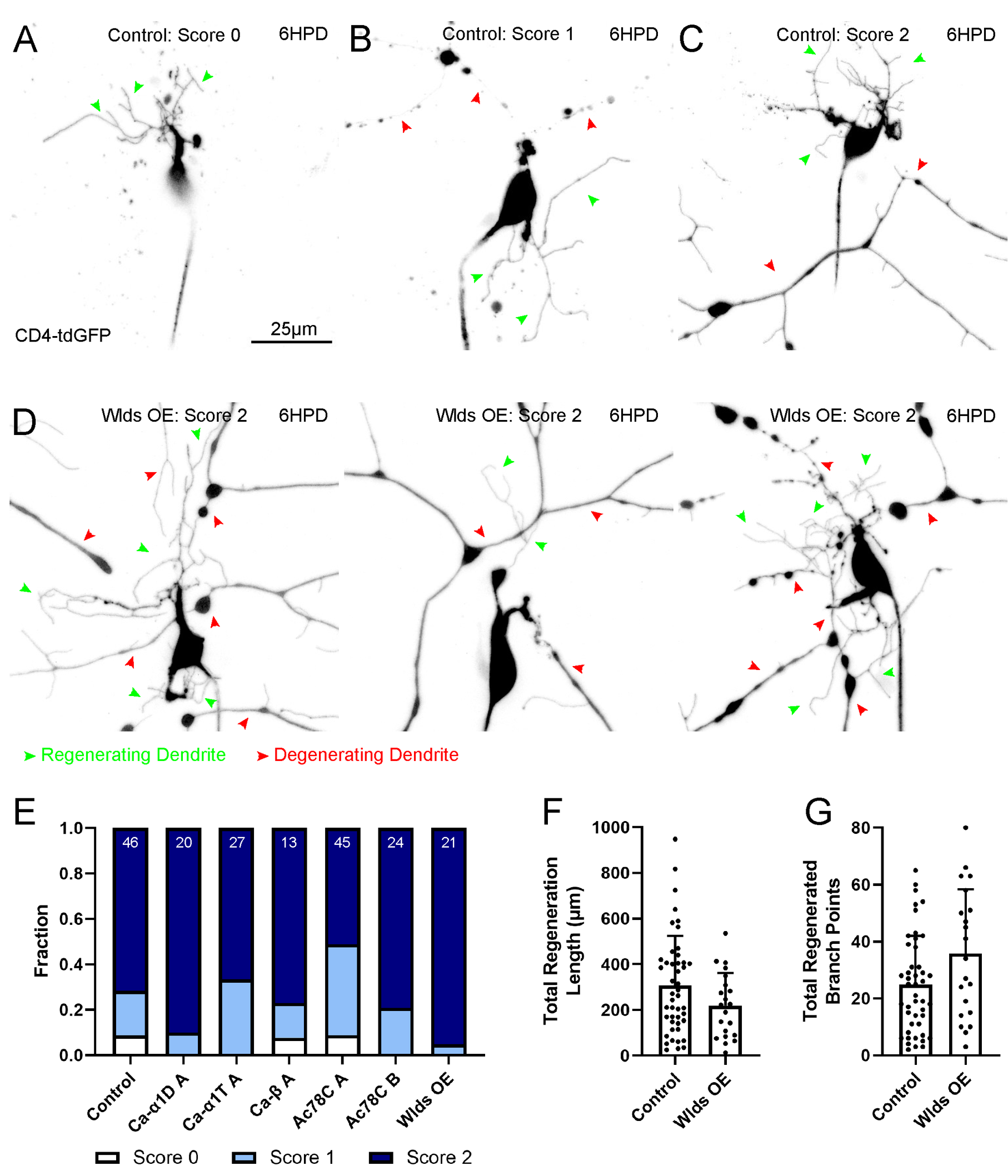

Supplement: S7 Fig — Example images of control neurons 6 hours after dendrite arbor removal (HPD) are shown (A-C) to demonstrate different degeneration scores: (A) score 0—no trace of degenerating dendrites, (B) score 1- degenerating dendrites still observed but without continuous regions, and (C) score 2 -degenerating dendrites observed with continuous regions present. (D) Example neurons expressing the Wallerian degeneration slow (Wlds) protein are shown at 6 HPD. Note that some newly grown dendrites seem to overlap the degenerating dendrites. Degeneration scores for all genotypes assayed at 6 HPD are summarized in (E). Numbers on the bars are numbers of cells analyzed for each condition. Total regeneration length and total regenerated branch points 6HPD are quantified in (F) and (G) and both are compared with a Mann-Whitney test. Control data is the same set shown in Fig 7D and 7E. No significant difference was found between control and Wlds OE. Error bars are standard deviations. (TIF) [file pgen.1011388.s007.tif]

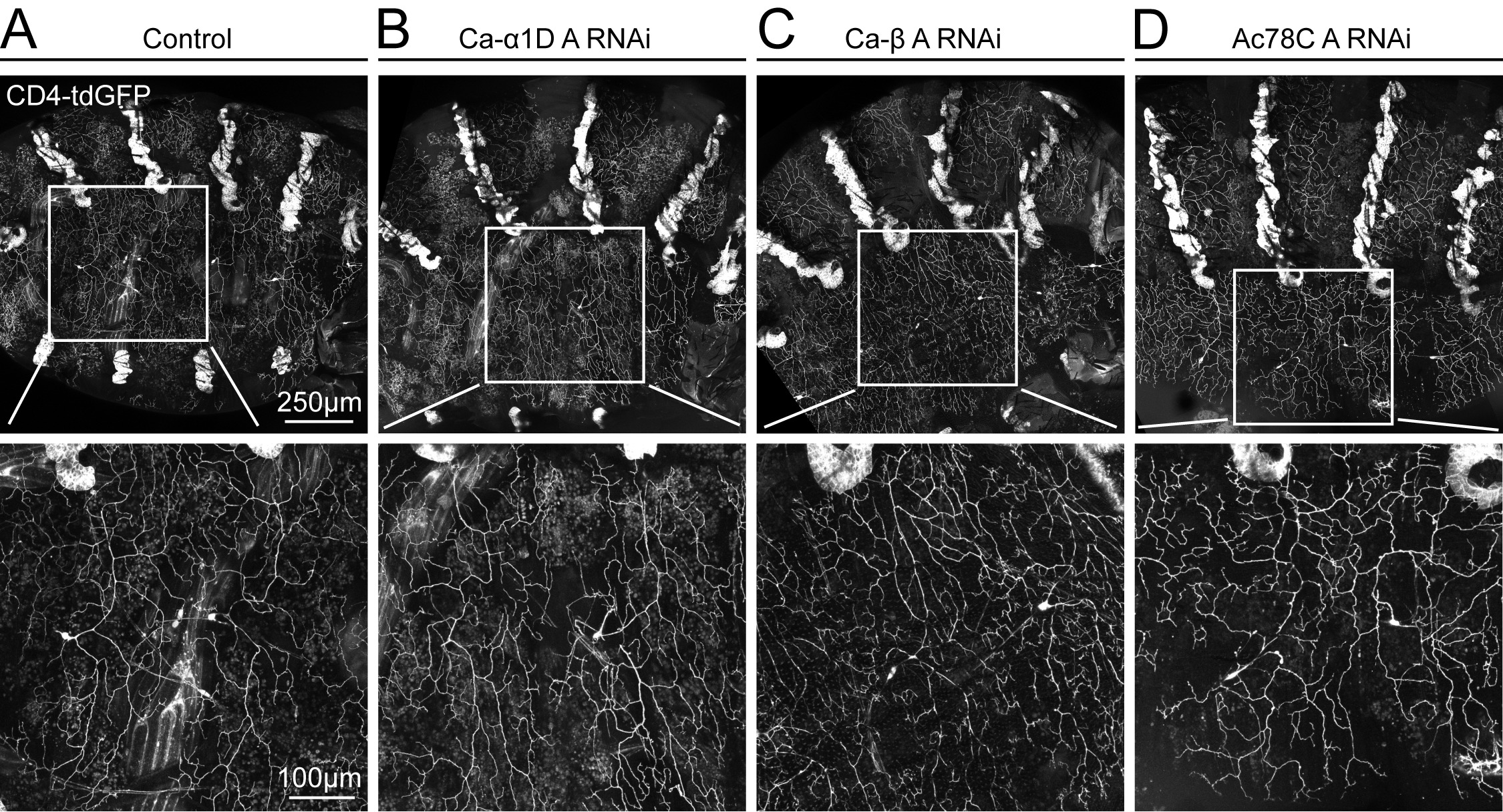

Supplement: S8 Fig — Shown are example overviews of uninjured v’ada neurons in abdomens of adult Drosophila expressing control (A), Ca-α1D (B), Ca-β (C), and Ac78C (D) RNAi. White square denotes area magnified in second row of images. No obvious defects in tiling or complexity are observed. (TIF) [file pgen.1011388.s008.tif]
