# Supplementary material for: Voltage-gated calcium channels act upstream of adenylyl cyclase Ac78C to promote timely initiation of dendrite regeneration
Source: PLoS Genet. 2024 Aug 26;20(8):e1011388. doi: 10.1371/journal.pgen.1011388 (PMC11379402; doi:10.1371/journal.pgen.1011388)
Supplement: S1 Methods — This file includes a description of the additional methods used to acquire data presented in the Supplemental Figures. (DOCX) [file pgen.1011388.s011.docx]

**Supplemental Methods**

**“Explosion” cuts to monitor GCaMP6f saturation**

For the explosion cut experiment, we turned the UV laser power up until it was high enough to produce a cavitation bubble and acquired images for 10-20 seconds after the cut. We are unable to quantify average fluorescence intensity in the cell body over time, as the cavitation bubble causes severe damage to the neuron and surrounding tissues, causing the neuron cell body to float and contort. The “Maximum post-cut fluorescence intensity” quantified in Figure S1 is the value of the brightest pixel in the cell body at any point after the explosion cut. Similarly, the values for “soft” axon and dendrite cut are the brightest pixel, not the highest average of the whole soma.

**GCaMP6f distal vs proximal cuts assay**

Experiments were performed identically to previous GCaMP6f live assays, except the cut point was at various distances from the soma. While actual distance varied, approximate delegations are <10, 40, and 80 microns from the soma for dendrite cuts and <10, 20, and 40 microns for axon cuts. Quantification was performed the same as previous experiments.

**GCaMP6f single vs full cut assay**

For Figure S2 A-B, cuts were performed slightly differently than in previous figures. A single cut is simply cutting one dendrite. A full cut is cutting all dendrites on a specific neuron. For full cuts, we used neurons with 3 or 4 dendrites to limit time spent cutting. Precision full cuts are not possible while imaging as the microscope did not have a motorized stage, thus requiring this modified method. For single cuts, one dendrite was cut while looking through microscope eyepieces, and then view was switched to microscope software for image acquisition. For full cuts, all dendrites were cut while looking through eyepieces, and then images were acquired. An image of the neuron was taken before cutting in both cases. Both of these conditions contain a lag time between first cut and image start which is reflected in the y value at t=0 of Figure S2B. All data points are normalized to their respective pre-image.

**GCaMP in ER lumen assay**

Flies with 17xUAS-ER-GCaMP-210 were acquired from BDSC (stock #91397) and crossed to females of the ppk:Gal4 tester line. Injury assays were performed and quantified identically to other GCaMP assays on scope #3. For soma cut condition, the cutting laser was placed over the cell body (not nucleus) and the MicroPoint laser was applied as if cutting dendrites or axons.

**Categorization of GCaMP6f pattern of increase**

To categorize how fluorescence increases spread across the cell body, the first few frames after injury were qualitatively assessed and binned into 3 categories: no increase, wave increase, or global increase. Videos where there was no fluorescence increase were binned as “no increase”; videos where the fluorescence intensity in the soma increased uniformly were binned as “global increase”; videos where fluorescence intensity increased in an asymmetric manner (getting brighter near the cut side of the cell first, then spreading across the rest of the soma) were binned as “wave increase.”

**Dendrite degeneration scoring**

6HPD images were scored for remaining pieces of cut off dendrites by an individual blind to genotype; the scorer was not involved in data collection. Each image was qualitatively classified into one of the three levels of dendrite degeneration: score 0- no trace of degenerating dendrites, score 1- degenerating dendrites still observed but without continuous regions, and score 2- degenerating dendrites observed with continuous regions present.

**Adult v’ada imaging**

Adult flies (eclosed within last 24h) were knocked out with CO2 and had their wings and rear legs removed. They were then placed on their side in a drop of glycerol on a microscope slide. A coverslip was taped on top. The abdomens were imaged on 10x and 20x objectives on the LSM800 inverted confocal microscope.
